# Genetic Variability of Human Cytomegalovirus Clinical Isolates Correlates With Altered Expression of Natural Killer Cell-Activating Ligands and IFN-γ

**DOI:** 10.3389/fimmu.2021.532484

**Published:** 2021-04-09

**Authors:** Ganna Galitska, Alessandra Coscia, Diego Forni, Lars Steinbrueck, Simone De Meo, Matteo Biolatti, Marco De Andrea, Rachele Cagliani, Agata Leone, Enrico Bertino, Thomas Schulz, Angela Santoni, Santo Landolfo, Manuela Sironi, Cristina Cerboni, Valentina Dell’Oste

**Affiliations:** ^1^ Laboratory of Pathogenesis of Viral Infections, Department of Public Health and Pediatric Sciences, University of Turin, Turin, Italy; ^2^ Neonatal Unit, Department of Public Health and Pediatric Sciences, University of Turin, Turin, Italy; ^3^ Laboratory of Bioinformatics, Scientific Institute IRCCS E. Medea, Bosisio Parini, Italy; ^4^ Institute of Virology, Hannover Medical School, Hannover, Germany; ^5^ Laboratory of Molecular Immunology and Immunopathology, Department of Molecular Medicine, “Sapienza” University of Rome, Rome, Italy; ^6^ Center for Translational Research on Autoimmune and Allergic Disease - CAAD, University of Piemonte Orientale, Novara, Italy

**Keywords:** human cytomegalovirus (HCMV), innate immunity, congenital infection, next generation sequencing, genetic variability, multiple-strain infection, NK cells, immunomodulation

## Abstract

Human cytomegalovirus (HCMV) infection often leads to systemic disease in immunodeficient patients and congenitally infected children. Despite its clinical significance, the exact mechanisms contributing to HCMV pathogenesis and clinical outcomes have yet to be determined. One of such mechanisms involves HCMV-mediated NK cell immune response, which favors viral immune evasion by hindering NK cell-mediated cytolysis. This process appears to be dependent on the extent of HCMV genetic variation as high levels of variability in viral genes involved in immune escape have an impact on viral pathogenesis. However, the link between viral genome variations and their functional effects has so far remained elusive. Thus, here we sought to determine whether inter-host genetic variability of HCMV influences its ability to modulate NK cell responses to infection. For this purpose, five HCMV clinical isolates from a previously characterized cohort of pediatric patients with confirmed HCMV congenital infection were evaluated by next-generation sequencing (NGS) for genetic polymorphisms, phylogenetic relationships, and multiple-strain infection. We report variable levels of genetic characteristics among the selected clinical strains, with moderate variations in genome regions associated with modulation of NK cell functions. Remarkably, we show that different HCMV clinical strains differentially modulate the expression of several ligands for the NK cell-activating receptors NKG2D, DNAM-1/CD226, and NKp30. Specifically, the DNAM-1/CD226 ligand PVR/CD155 appears to be predominantly upregulated by fast-replicating (“aggressive”) HCMV isolates. On the other hand, the NGK2D ligands ULBP2/5/6 are downregulated regardless of the strain used, while other NK cell ligands (i.e., MICA, MICB, ULBP3, Nectin-2/CD112, and B7-H6) are not significantly modulated. Furthermore, we show that IFN-γ; production by NK cells co-cultured with HCMV-infected fibroblasts is directly proportional to the aggressiveness of the HCMV clinical isolates employed. Interestingly, loss of NK cell-modulating genes directed against NK cell ligands appears to be a common feature among the “aggressive” HCMV strains, which also share several gene variants across their genomes. Overall, even though further studies based on a higher number of patients would offer a more definitive scenario, our findings provide novel mechanistic insights into the impact of HCMV genetic variability on NK cell-mediated immune responses.

## Introduction

Human cytomegalovirus (HCMV) is a widespread pathogen persisting in over half of the human population (1). The clinical symptoms of HCMV infection vary greatly from one individual to another, ranging from mild asymptomatic infections in healthy adults to severe life-threatening syndromes in immunodeficient patients, elderly, and congenitally infected newborns (2). Since neither vaccines nor effective therapeutics against HCMV are currently available, there is an urgent unmet clinical need to address HCMV infection in these particularly vulnerable patients (3–5).

Throughout evolution, HCMV has been subjected to intense selective pressure from the innate immune system (6–9). Among lymphocytes, NK cells play a crucial role in controlling HCMV infection early at the onset of infection, when they rapidly detect and lyse HCMV-infected cells through natural cytotoxic activity and/or antibody-dependent cell-mediated cytotoxicity (ADCC) (10–13). Moreover, NK cells exert their protective functions through cytokine and chemokine secretion and are capable of killing target cells *via* the TNF superfamily of ligands (14–17). NK cell target recognition, activation, and effector functions are regulated by a plethora of signals acting on numerous activating and inhibitory NK receptors, such as NKG2D and DNAM-1/CD226 (18, 19). Thus, it is conceivable that HCMV might have learned escape strategies from NK cell-mediated immune surveillance in order to establish a successful life-long persistence in the host. As a result, a delicate equilibrium between HCMV infection and innate immunity ultimately determines the outcome of infection (20, 21).

In this scenario, some peculiar characteristics of HCMV are particularly important. First, its large dsDNA genome (~235 kb) not only boasts 165 canonical ORFs (22–24) but also encodes multiple alternative transcripts and engages noncanonical translation initiation sites (25–28). Another peculiarity is represented by the increased genome encoding capacity of HCMV—this virus encodes a myriad of genes whose functions are currently unknown—probably confers a survival advantage to the virus against the host innate immune response, a hypothesis supported by the growing number of HCMV genes directly involved in NK cell modulation (12). A third important characteristic of HCMV is its high genetic variability, contradicting the expectation that, being a large double-stranded DNA virus, it should retain high-level genome stability (29). Of note, HCMV genetic variations have been detected particularly in genes contributing to immune evasion (30). Furthermore, genetic diversity within a single host could be partly explained by high-frequency multiple strain co-infection (mixed infection), *de novo* mutations, and reactivation of the latent virus (31).

Even though the prevalence of intra-host HCMV diversity has initially been attributed to the early occurrence of *de novo* mutations (32, 33), recent data suggest that it may be the result of mixed infection with genetically diverse HCMV strains (31, 34) and extensive recombination (30, 34, 35). Many of these genetic alterations may ultimately affect cell tropism and evasion from innate and adaptive defenses. Thus, understanding the contribution of mixed infection and recombination to viral diversity appears crucial to shed light on the mechanism of HCMV evolution, immune adaptation, and pathogenesis.

The present study aimed to determine whether the ability of HCMV clinical isolates to modulate NK cell responses would correlate with differences in their genetic composition. For this purpose, we chose to analyze clinical isolates instead of highly passaged laboratory strains to rule out the occurrence of adaptive HCMV mutants, as reported previously (23, 36–41). All five HCMV clinical isolates were obtained from pediatric patients with confirmed HCMV congenital infection, previously characterized as those displaying a high phenotypic heterogeneity (42). These isolates were then analyzed for genetic diversity across the entire HCMV genome by next generation sequencing (NGS) to establish a relationship between genetic variability and modulation of NK cell functions. Overall, our work highlights the importance of combining genome sequencing with immunological assays to determine the functional consequences of genetic variations of HCMV clinical isolates.

## Materials and Methods

### Cells and Viruses

Primary human foreskin fibroblasts (HFFs, American Type Culture Collection, ATCC SCRC-1041™) were cultured in Dulbecco’s Modified Eagle’s Medium (Sigma-Aldrich, Milan, Italy) supplemented with 10% FCS (Sigma-Aldrich, Milan, Italy) according to ATCC specifications. HCMV clinical isolates derived from urine samples were obtained from a previously characterized cohort of pediatric patients (42). The urine samples were directly inoculated in HFFs to boost the infected cell population. The isolates were then propagated until approximately 60% of cells showed a cytopathic effect. All isolates were used before passage 3 to avoid cell culture adaptation (43, 44). The HCMV strain Merlin was kindly provided by Gerhard Jahn and Klaus Hamprecht (University Hospital of Tübingen, Germany), then propagated and titrated on HFFs by standard plaque assay. For RT-qPCR, HFF-NK cell cocultures, and FACS experiments, at day 0, HFFs infected with different HCMV clinical isolates were stained intracellularly and analyzed by flow cytometry to measure the percentage of immediate early (IE) IE1/IE2 antigen positive cells (MAB810X; Merck Millipore, Burlington, USA). Subsequently, uninfected (mock) HFFs were co-cultured with infected HFFs at a ratio of 9:1. At the indicated times post-infection, HFFs were harvested and analyzed.

The study was approved by the Research Ethics Committee of the University Hospital of Turin “A.O.U. Città della Salute e della Scienza di Torino – A.O. Ordine Mauriziano – A.S.L. TO1” (No. 007816). Informed consent was obtained from parents of all study participants prior to collection of clinical data and biological samples. The study has been carried out in accordance with the Declaration of Helsinki.

### RNA Extraction, Retrotranscription, and RT-qPCR Analysis

Total RNA was extracted using TRI Reagent^®^ solution (Life Technologies, Carlsbad, USA) according to the manufacturer’s instructions. Total RNA (1 μg) was used for cDNA synthesis in a reaction volume of 20 µl using the Revert-Aid H-Minus FirstStrand cDNA Synthesis Kit (Thermo Fisher Scientific, Waltham, USA). Comparison of mRNA expression between samples (i.e., infected *vs.* mock) was performed by SYBR green-based RT-qPCR on an Mx3000P apparatus (Stratagene, San Diego, USA). Relative levels of each mRNA species were calculated using the 2^-ΔΔCt^ method with reference to the housekeeping gene glyceraldehyde-3-phosphate dehydrogenase (GAPDH). The following primers were used: MICA (Fw: 5’-GGGCTGACCATCCAGATGTA-3’; Rev: 5’-ATCTTCCCTTTTGCACCTCC-3’); MICB (Fw: 5’-AACCCTGACTGCACAGATCC-3’; Rev: 5’-GGTCCTGCTGTTTCTGGC); ULBP1 (Fw: 5’-AGGCCTTGAACTTCACACCA-3’; Rev: 5’-GCTTCTGCACCTGCTGTCT-3’); ULBP2/5/6: (Fw: 5’-CGTGGTCCAGGTCTGAACTT-3’; Rev: 5’-CAAGATCCTTCTGTGCCTCC-3’); ULBP3 (Fw: 5’-ATTCTTCTGATCCACCTGGC-3’; Rev: 5’-TCCGTACCTGCTATTCGACTG-3’); PVR/CD155 (Fw: 5’-TCCAATTATAGCCTGTGGGC-3’; Rev: 5’-GCTGCTGACTGTGAACCTCA-3’); B7-H6 (Fw: 5’-TCTCTTTCATGCCCACTTGA-3’; Rev: 5’- GCTGGAGGAAGCAGGAGAGT); GAPDH: (Fw: 5’-AGTGGGTGTCGCTGTTGAAGT-3’; Rev: 5’-AACGTGTCAGTGGTGGACCTG-3’).

### Antibodies and Reagents

The following PE-conjugated monoclonal antibodies (mAbs) were used in flow cytometry: anti-MICA (clone 159227), MICB (clone 236511), ULBP1 (clone 170818), ULBP2/5/6 (clone 165903), ULBP3 (clone 166510), B7-H6 (clone 875001), Nectin-2/CD112 (clone 610603) (all from R&D Systems, Minneapolis, USA), PVR/CD115 (clone SKII.4; BioLegend, San Diego, USA) and mouse control IgG (from BD). Anti-IFN-γ;, mouse control IgG (both from BD Biosciences, San Jose, USA), and anti-HLA-I (clone W6/32; Biolegend) were APC-conjugated. Other antibodies and reagents used were as follows: Alexa Fluor 488–conjugated anti-IE viral antigens (MAB810X; Merck Millipore, Burlington, USA); PE-conjugated anti-CD56, FITC-conjugated anti-CD3, APC-H7-conjugated Zombie NIR™ viability kit (BioLegend, San Diego, USA); PMA, ionomycin, brefeldin A and DMSO (all from Sigma-Aldrich, Milan, Italy).

### Immunofluorescence and FACS Analysis

Mock-infected or infected cells were harvested at the indicated day post-infection (dpi) and stained with specific mAbs. The mean fluorescence intensity (MFI) value of the isotype control IgG was subtracted from the MFI relative to each molecule. For intracellular staining of IE1/IE2 antigens, cells were fixed in 1% formaldehyde, permeabilized with 70% ethanol, and then incubated with Alexa Fluor 488-conjugated anti-IE mAb (MAB810X; Merck Millipore, Burlington, USA). Cells were acquired with a FACSCanto II flow cytometer (BD Biosciences, San Jose, USA) and analyzed with FlowJo 10 (ver. 10.0.7) software.

### NK Cell Cultures and IFN-γ Production

Peripheral blood polyclonal NK cells, obtained from healthy donors, were generated as previously described and used at 80-95% purity (45). HFFs were plated as described above and, at 2 dpi, NK cells were added to the wells at an NK : HFF ratio of 2:1. Positive and negative controls were obtained by culturing NK cells alone in the presence or absence of PMA (50 ng/ml) plus ionomycin (500 ng/ml), respectively. At the same time, brefeldin A was added to all wells at a concentration of 5 µg/ml. After an NK : HFF co-culture of 18 h, NK cells were harvested, and extracellular staining was performed using a mixture of FITC-conjugated anti-CD3, PE-conjugated anti-CD56 and APC-H7-conjugated Zombie NIR™ viability dye. After washing, cells were fixed, permeabilized, stained with the APC-conjugated anti-IFN-γ; mAb, and analyzed by flow cytometry.

### DNA Extraction and Next-Generation Sequencing

DNA was extracted from infected fibroblasts using Qiagen Blood Mini Kit (Qiagen, Hilden, Germany) and eluted in 60 µl PBS. Total DNA (500 ng) was used as input for library preparation. For Library preparation, an NEB Ultra II FS Kit (New England Biolabs, Ipswich, USA) was used, which provides a transposase-based fragmentation. Index sequences were added by 3 cycles of PCR with NEB NEBnext Index primers (New England Biolabs, Ipswich, USA). The libraries were loaded with a final concentration of 12 pM on a MiSeq (Illumina, San Diego, USA), using an Illumina MiSeq Reagent Kit v3 (600-cycles) with 11% Flow cell coverage for each sample. The resulting reads were trimmed to remove adaptor sequences and low-quality bases (q < 20) and mapped to the human genome (hg19) with bowtie2_2.2.2. Mapped reads were then discarded. The unmapped reads were used for *de novo* assembly with SPAdes_3.10.1 and CLC 10.0.1(Qiagen, Hilden, Germany) *de novo* Assembler. The contig sequences from CLC and SPAdes were combined and used for scaffolding in CLC. All samples were scaffolded to the Merlin Reference AY446894 from the GenBank. A draft assembly was constructed by extracting the consensus sequence from scaffolding. Subsequently, the sequences underwent several steps of error-correction and genome polishing. In a first step, GapFiller_1.10 was used to close gaps in the draft assembly, followed by remapping with bowtie2 and error correction by Pilon_1.22, using the quality trimmed and human filtered reads from the initial steps. The Remapping/Pilon process was performed in three iterations. The polished sequences were then aligned to the mapping-consensus from reference mapping using the MAFFT plugin in Geneious_11.1.5. The remaining gaps in the assembly were filled after alignment from the mapping consensus. To finish the assembly, a last error correction step was performed with a homemade script, which replaces nucleotides with higher probability. This script follows the “Best Practices” for variant calling, as recommended by GATK (Broad Institute), and uses several tools from GATK3. Annotations were transferred in Geneious with the tool “Annotate from…”. The threshold for annotation was a similarity of > 75%. The final low frequency variant calling was performed in CLC after mapping the deduplicated reads back to the assembled genome. To remove sequencing errors from the result tables, the variants were filtered for a minimum mapping quality of 20, no homopolymer stretches, and a forward/reverse balance of > 0.3.

For genotyping, the reads were mapped to the reference sequences of hypervariable genes from different HCMV strains, which represent different genotypes. For plotting variant frequency and binned variant frequency, a homemade R-script from Elias Haage was used. Also, all final sequences were aligned to the Merlin reference. To determine all differences with the Merlin-reference sequence, single alignments of the final assembled sequences with the Merlin Ref were performed in Geneious, using the MAFFT plugin. By using the function “Find Variations/SNPs”, tables of all differences were created and exported. All mappings were performed in CLC, with a landing fraction of 50% and a similarity fraction of 80%. Complete genome sequences of all HCMV isolates were submitted to GenBank under the following accession numbers: P4: MT070138; P6: MT070139; P10: MT070140; P14: MT070141; P15: MT070142.

### Alignments, Neighbor-Net Split Network, and Similarity Scores

Whole genome sequence alignments were generated using MAFFT (46) with the default parameters. A neighbor-net split network of all whole-genome sequences was constructed with SplitsTree v4.13.1 (47) using uncorrected p-distances and all polymorphic sites, after removing gap sites. Pairwise identity scores were calculated as 1-(M/N), where M is the number of mismatching nucleotides, and N is the total number of positions along the alignment at which neither sequence has a gap character, as previously reported (48).

### Statistical Analysis

Statistical tests were performed using GraphPad Prism version 5.00 for Windows (GraphPad Software, San Diego California USA), unless specified differently in the text. The data were presented as means ± standard deviations (SD). Means between one or two groups were compared by using a two-way analysis of variance with Bonferroni’s post-test or paired t-test. Differences were considered statistically significant for *P < 0.05; **P < 0.01; ***P < 0.001.

## Results

### HCMV Clinical Strains Differently Modulate NK Cell Activating Ligands

We previously demonstrated that HCMV clinical strains from urine samples of congenitally infected patients displayed high levels of phenotypic variability alongside high genetic variation in regions responsible for immunomodulation (42). Thus, we asked whether these differences would influence the ability of HCMV to modulate the host immune response. For this purpose, we determined the expression levels of various ligands of the NK cell-activating receptors NKG2D, DNAM-1/CD226, and NKp30 (6, 16) in HFFs infected with five selected clinical isolates (hereinafter referred to as P4, P6, P10, P14, and P15). The selection of these clinical strains was based on their phenotypic characteristics in different cell culture models (i.e., HFFs, HUVECs, and ARPE-19 cells) (42). Specifically, the replication of cell-associated isolates was quantified by focus expansion assay (FEA), as previously described (42). Based on FEA results, P14 and P15 were fast-replicating, syncytial-forming strains (referred to as “aggressive”), while P4 was “moderate”, and P6 and P10 slow-replicating (“non-aggressive”) (42). Upon infection, HFFs were co-cultured with an excess of uninfected HFFs (mock) (ratio of 9:1) for different time points post-infection and then subjected to RT-qPCR and FACS analysis to test mRNA and protein expression levels of selected NK cell ligands. The infection rate was calculated by intracellular staining of IE1/IE2 antigens (**Supplementary Figures 1A, B**).

**Figure 1:**
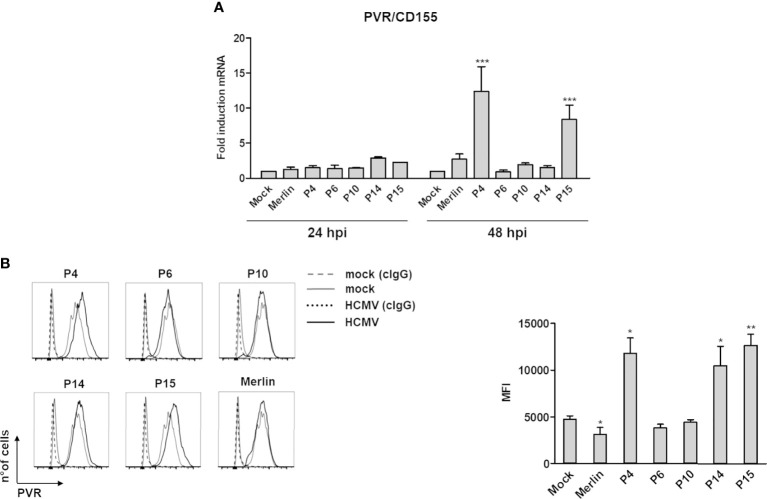
Modulation of the NK cell ligand PVR/CD155 by HCMV clinical isolates. **(A)** Primary human foreskin fibroblasts (HFFs) infected with the indicated clinical isolates (P4, 6, 10, 14, and 15), the Merlin strain, or uninfected (mock) were co-cultured with an excess of uninfected HFFs, as described in Materials and Methods, and subjected to RT-qPCR to measure PVR/CD155mRNA expression levels. Values were normalized to the housekeeping gene glyceraldehyde-3-phosphate dehydrogenase (GAPDH) mRNA and plotted as fold induction relative to mock-infected cells (set at 1). Data from three experiments performed at 24 and 48 hours post-infection (hpi) are shown. Error bars show standard deviation (SD) (***P < 0.001; two-way ANOVA followed by Bonferroni’s post-tests, for comparison of infected *vs.* mock cells). **(B)** FACS analysis assessing PVR/CD155 expression at 3 days post-infection. *Left panel:* a representative experiment of at least four performed with all HCMV isolates is shown. Dashed and dotted lines indicate isotype control in mock or HCMV-infected cells, respectively. *Right panel:* data derived from at least four experiments performed with all HCMV isolates. PVR/CD155 expression levels are presented as mean fluorescence intensity (MFI) ± SE (*P < 0.05; **P < 0.01, paired Student *t* test for comparison of infected *vs.* mock cells).

As shown in **Figure 1**, the phenotypically “aggressive” P15- and “moderate” P4-infected HFFs showed enhanced levels of the DNAM-1/CD226 ligand poliovirus receptor (PVR/CD155) mRNA at 48 but not 24 hours post infection (hpi) in comparison with Merlin- or mock-infected cells, while infection with P6, P10, and P14 did not lead to significant changes in PVR/CD155 expression at either time point (**Figure 1A**). Consistent with the RT-qPCR results, P4 and P15 upregulated PVR/CD155 cell surface protein expression (**Figures 1B**) at 3 days post-infection (dpi). Intriguingly, also the second aggressive isolate P14, which failed to upregulate PVR/CD155 mRNA at both 24 and 48 hpi, increased PVR/CD155 protein expression, suggesting that additional posttranslational events were probably needed for this variant to trigger maximum ligand induction. Furthermore, we have observed a significant mRNA induction of the NKG2D receptor ligand MICA in P4- or P15-infected cells at 48 hpi, which have not however been confirmed at the protein level (**Supplementary Figure 2A**). Likewise, ULPBs (i.e., ULBP2/5/6 and 3) were upregulated at the mRNA level upon infection with some strains— ULBP2/5/6: Merlin, P4, P14, P15 at 24 hpi, and Merlin and P15 at 48 hpi; ULBP3: P4, P6, and P10 at 24 hpi, and Merlin and P4 at 48 hpi—, but their cell surface expression has been mostly downregulated or unaffected in comparison with mock-infected cells (**Supplementary Figures 2B, C**). Finally, the other NKG2D and DNAM-1/CD226 ligands MICB, and Nectin-2/CD112, as well as the NKp30 ligand B7-H6 were undetectable in both mock- or HCMV-infected cells (data not shown).

Altogether, these results strict display no correlation between isolate aggressiveness and activating ligand expression, and at the cell surface they appeared to be - in general - down-regulated. However, an exception to such pattern has been observed for PVR/CD155 in cells infected with three different isolates.

### Functional Activity of NK Cells Co-Cultured With HFFs Infected With Different HCMV Clinical Isolates

**Figure 2:**
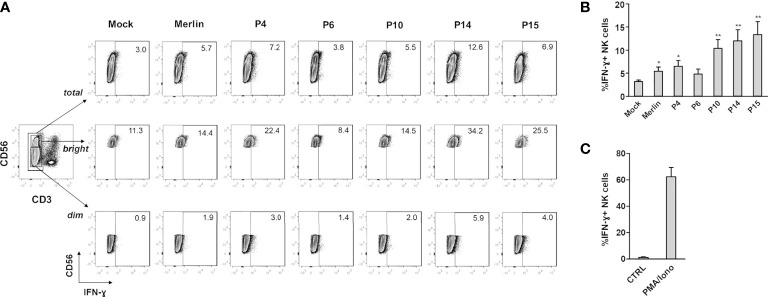
IFN-γ expression in NK cells co-cultured with HCMV-infected HFFs. NK cells were co-cultured with mock- or HCMV-infected HFFs at day 2 post infection, as described in Materials and Methods. The day after, NK cells were harvested and stained for intracellular IFN-γ. **(A)** A representative experiment of at least four performed with all HCMV isolates is shown. Numbers indicate the percentage of IFN-γ^+^ cells in the gate of CD3-CD56^+^ (total), CD3-CD56dim (dim), or CD3-CD56bright (bright) NK cells. All cells were first gated among viable (Zombie-) population. **(B)** Cells were analyzed as in panel **(A)**, and data are expressed as the mean percentage (%) ± SE of IFN-γ+ cells in the gate of total CD3-CD56^+^ NK cells. Data are from at least four independent experiments (*P < 0.05; **P < 0.01 paired Student *t* test for comparison of infected *vs.* mock cells). **(C)** Negative (ctrl) and positive (PMA/iono) controls for IFN-γ production are also shown, and are referred to NK cells cultured alone or in the presence of PMA plus ionomycin.

To determine whether the observed modulation of NK cell-activating ligands by selected HCMV clinical strains resulted in differences in NK cell functional activity, we analyzed IFN-γ; expression by NK cells co-cultured with HFFs infected with different HCMV isolates (**Figure 2**). Indeed, IFN-γ secretion in NK cells upon HCMV infection is known to limit HCMV replication by triggering a Th1 response and promoting cell resistance to infection, *via* the so-called “anti-viral state” (49, 50). HFFs infected with clinical isolates were co-cultured with an excess of uninfected HFFs, as described above, and at 2 dpi, NK cells were added and co-cultured with HFFs at a ratio of 2:1, in the presence of brefeldin A. After 18 h of NK : HFF co-culture (3 dpi in total), NK cells were harvested and IFN-γ; expression was analyzed by intracellular staining on CD3-CD56^+^ NK cells (**Figure 2A**). As a positive or negative control, NK cells were cultured alone in the presence or absence of PMA plus ionomycin, respectively (**Figure 2C**). We observed a greater percentage of NK cells capable of producing IFN-γ in response to infection with the “aggressive” strains P14 and P15, and to a lesser extent with the “moderate” P4 and “non-aggressive” P10 (**Figures 2A, B**). By gating on CD3-CD56dim or CD3-CD56bright NK cells, the highest percentage of IFN-γ^+^ NK cells seemed to be confined to the CD56bright population, as previously shown for this time interval after stimulation (>16 h) (51, 52).

Finally, to understand if IFN-γ; modulation was a consequence of HLA-I expression, we tested it together with IE1/IE2 viral protein expression by double-staining followed by FACS analysis. As shown in **Supplementary Figure 2D**, HLA-I was strongly downregulated in Merlin-, P4-, P14-, P15-infected cells, where most of the cells were infected (80-90% of IE+ cells, as indicated). On the other hand, P6 showed HLA-I downregulation only in IE+ infected cells, but not in uninfected cells, while P10 had an intermediate phenotype, indicating that triggering of IFN-γ; production was not a consequence of the sole HLA-I expression.

Altogether, these results demonstrate that clinical isolates differ in their ability to modulate NK cell-activating ligands and NK cell effector functions.

### NGS Assessment of HCMV Genetic Variability in HCMV Clinical Isolates

To determine whether the differences observed in phenotypic features (42) and immunological response could be primarily ascribed to differences in genetic composition, we employed an NGS approach to sequence the HCMV clinical isolates.

**Figure 3:**
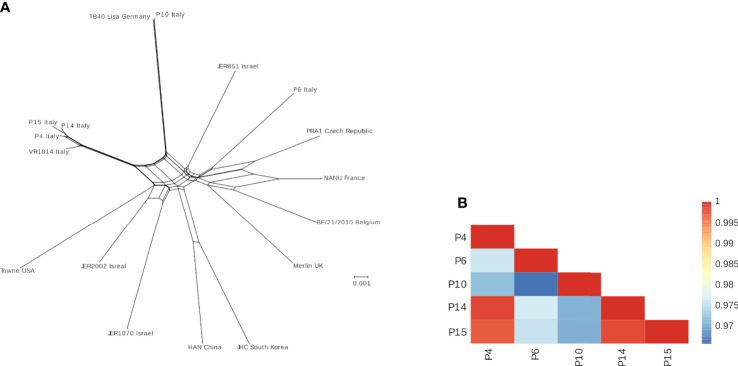
**(A)** Neighbor-net split network of the five HCMV isolates plus additional genomes sequences. The geographic location where each sample was isolated is indicated. **(B)** Color-coded pairwise identity matrix for the five isolates. Each cell represents the percentage identity score between two sequences (indicated horizontally and vertically). The legend indicates the correspondence between pairwise identities and the color code. Identity scores were computed over all positions where gaps are not observed in either sequence.

Sufficient coverage has been obtained for all isolates (**Supplementary Table 1**). Analysis of the frequency and distribution of heterozygous variants across these genomes suggested that all isolates derived from single-strain infections, as most of such variants were either low frequency or unevenly distributed (**Supplementary Figure 3**). P14 and P15 showed clustering of high-frequency variants in specific regions corresponding to the UL20-UL24 and UL48 genes (**Supplementary Figure 3**). For all isolates, a consensus genome has been obtained and aligned with other available sequences from urine samples and laboratory strains to generate a neighbor-net split network (**Figure 3**), a method that allows inference of evolutionary relationships in the presence of conflicting phylogenetic signals (e.g., recombination). In line with previous reports (35), no evidence of geographic clustering for HCMV isolates was observed. However, isolates P4, P14, and P15 are closely related among themselves and with strain VR1814, originally isolated in Italy in 1996 (53) (**Figure 3A**). Indeed, the average identity among these sequences appear higher than 99% (**Figure 3B**).

Since gene-disrupting mutations are known to be common in HCMV clinical isolates, irrespective of cell culture adaptation (35), the five isolates were analyzed for the presence of mutations disrupting their coding potential (**Table 1**). Genes that are commonly disrupted in clinical isolates (*RL6*, *RL13*, *UL1*) (54) were indeed found to be mutated in all samples, except for the P6 strain. Interestingly, a deletion involving the *UL144-UL140* region has been detected in the three closely related isolates P4, P14, and P15 (**Table 1**). Furthermore, alignment of the predicted protein sequences of molecules known to modulate NK cell affecting it in either inhibitory way (i.e., UL18, RL11, UL119, UL135, and UL83), activating (i.e., UL16, UL141, UL142, UL148, US12, US14, US9, US18, US20, UL16, and UL142), or both (i.e., UL40), showed very few differences among the isolates, and most changes were contributed by P10 (**Supplementary Figure 4**). Clearly, this is only the case of genes not involved in deletion events (i.e., UL140-UL144).

**Table 1:** Genes containing ORF-disrupting mutations.

Isolate	Event	Genes
**P4**	Deletion (1053 bp)	*RL13*-*UL1* (partial)
	Deletion (3173 bp)	*UL144* (partial), *UL142*, *UL141*, *UL140* (partial)
	Complex event^1^	*IRS1^1^*
		
**P6**	ND^2^	ND^2^
		
**P10**	Deletion (381 bp)	*RL6*
	Complex event (duplication/deletion)	*US34* (dup), *US34A* (dup), *US33A* (dup); *US3* (del);
		
**P14**	Deletion (1053 bp)	*RL13*-*UL1* (partial)
	Deletion (3173 bp)	*UL144* (partial), *UL142*, *UL141*, *UL140* (partial)
	Complex event^1^	*IRS1^1^*
		
**P15**	Deletion (1053 bp)	*RL13-UL1* (partial)
	Deletion (3173 bp)	*UL144* (partial), *UL142*, *UL141*, *UL140* (partial)
	Complex event^1^	*IRS1^1^*

^1^Due to the repetitive nature of the region, it was impossible to establish mutation events with certainty; ^2^None detected.

## Discussion

The worldwide spread of HCMV infection concomitant with the absence of an effective vaccine, reliable diagnostic methods, and safe therapeutics represents a significant challenge in HCMV disease management and prevention in various clinical settings, especially in congenital infections with unpredicted outcomes (55–60).

In recent years, many efforts have been directed towards understanding whether different clinical outcomes may be ascribed to alterations at the viral genome level (61, 62), which is supported by the observation that the outcome of infection is also dependent on the host immune status (63, 64).

More than half of the HCMV genome encodes proteins with immunomodulatory functions (24, 65), a large array of which can inhibit NK cell functions (13, 66). However, despite recent advances in the characterization of the HCMV genome, the functional relevance of HCMV genetic variability remains poorly understood.

Sanger sequencing and, more recently, high-throughput approaches have been extensively applied to assess HCMV genome variability (35, 67, 68), though no effort has been so far made to correlate the genome composition of HCMV clinical strains with its immunomodulatory activity. Thus, here we sought to determine whether the genetic variability among HCMV clinical isolates obtained from congenitally infected pediatric patients (42) would influence activating ligand expression and, consequently, NK cell effector functions.

Our *in vitro* results provide direct evidence that NK cell-activating ligands are differently modulated by HCMV clinical isolates. In particular, PVR/CD155 was strongly upregulated by the moderate-to-aggressive P4, P14, and P15 strains, whereas it was downregulated by the laboratory strain Merlin, in good agreement with previous works (69–71). While P4- and P15-mediated PVR/CD155 upregulation was already evident at the mRNA level at 48 hpi, the increased PVR/CD155 protein level could only be observed after 3 days of P14 infection, suggesting that additional posttranslational events may be involved in the regulation of this ligand. We think that there should not necessarily exist an indirect correlation between the expression of all activating ligands and “aggressiveness” (the more aggressive isolate, the fewer ligands expressed). At the same time, mRNA expression may not necessarily correlate with the expression of the encoded protein. Since HCMV is paradigmatic in its capability to down modulate all known NKG2D and DNAM-1 ligands, one would expect a generalized suppression of their expression, particularly at the protein level. However, the vast majority of HCMV ORFs influencing NK cell recognition of infected cells inhibit activating ligand cell surface expression more than their mRNA, as a widely acknowledged fact in the field. Thus, for all these reasons, it is not surprising that all the ligands tested were - in general - downregulated, independently from their mRNA expression. However, since PVR/CD155 appeared to be an exception, further investigations into the reason(s) behind its upregulation were carried out (**Figure 1**), as compared to previous studies, including those from us (71).

We asked whether this phenotype could be ascribed to specific genome alterations of these HCMV strains. Interestingly, NGS analysis revealed that these strains are phylogenetically related (**Figure 3**). Surprisingly, P4, P14, and P15 share a deletion in the UL141 gene, which normally inhibits the expression of PVR/CD155 expression to keep NK cells in check (69–71). Remarkably, the increased expression of PVR/CD155 in P4, P14, and P15-infected cells correlated with enhanced expression of IFN-γ by those NK cells cocultured with HFF infected with the same strains, suggesting a potential link between genotype, phenotype, and functional effects. Although it would be interesting to further investigate IFN-γ production by different NK cells subsets, and thus at different time-points after stimulation, our results were indeed unexpected, as one would assume that an “aggressive” strain would more readily escape NK cell control also *via* inhibition (and not stimulation) of this potent cytokine used by NK cells to limit HCMV replication. However, IFN-γ is also a well-known marker directly induced by the infection. For example, the QuantiFERON-CMV test relies on the measurement of IFN-γ release in order to identify patients at risk to develop CMV disease and is currently employed in the clinical practice (72).

Surprisingly, P10, even if classified as a “non-aggressive” isolate, induces a considerable proportion of IFN-γ+ NK cells, albeit lower while compared to the “aggressive” isolates P14 and P15. One possible explanation of such effect could be ascribed to the numerous mutations encountered in the sequences of P10 proteins involved in the evasion from NK cell recognition (**Supplementary Figure 4**).

It is well-established that pUL141 acts as a potent immune modulator that impairs surface expression of PVR/CD155 by sequestering it into the endoplasmic reticulum (ER) (69, 73, 74). Two other NK cell-associated host proteins, namely TNF-related apoptosis inducing ligand receptors 1 and 2 (TRAIL-R1 and R2), involved in the transmission of apoptotic signals and caspase activation, are also targeted by pUL141 and subjected to intracellular retention (75, 76). Moreover, pUL141 co-operates with another viral modulator (US2) to degrade Nectin-2/CD112, an additional DNAM-1/CD226 ligand, *via* the E3 ligase TRC8 and proteasomal degradation (70, 77). Hence, pUL141 prevents NK cell-mediated cytotoxicity by targeting at least four different proteins *via* two independent mechanisms (12). We, therefore, hypothesize that our moderate-to-aggressive HCMV isolates, due to a more error-prone and faster replication rate, may have lost immunoevasion genes, such as UL141, thereby becoming more susceptible to NK cell detection. Concomitantly, the fast-replication of these particular strains may result in more stochastic dissemination into the host, considering their broad *in vitro* growth patterns previously reported (42), which would render them more readily detectable by the immune system (e.g., cytotoxic lymphocytes and neutralizing antibodies). Conversely, the non-aggressive strains P6 and P10, with an intact UL141, did not significantly modulate the expression of PVR/CD155. However, despite the absence of UL141 mutations in its genome, P10 was able to elicit IFN-γ expression by NK cells. This could be ascribed to the numerous mutations detected in P10-encoded molecules associated with NK cell modulation, which may eventually result in enhanced IFN-γ secretion (**Supplementary Figures 3** **,** **4**).

The analysis of other NK cell-activating ligands revealed that ULBP2/5/6 was downregulated independently from the strain. In line with these results, no deletions have been detected in the HCMV gene UL16, previously shown to be involved in downregulating this ligand (45).

MICA, MICB, ULBP3, Nectin-2/CD112, and the NKp30 ligand B7-H6 were not significantly modulated by any of the HCMV isolates. This is quite surprising given that P4, P14, and P15 displayed a deletion in the UL142 gene, responsible for ULBP3 and MICA downregulation (78–80). However, the other HCMV ORFs known to inhibit the expression of these ligands (e.g., US9, US18, and US20) (81, 82) were not deleted or mutated, suggesting a potential compensatory role of these ligands.

Along with complete UL141 and UL142 gene loss in the P4, P14, and P15 strains, NGS analysis also revealed partial loss of UL144 and UL140. UL144 is a highly variable gene within clinical isolates (42, 83, 84), but to date no inhibitory function for UL144 on NK cells has been demonstrated (85). However, UL144 can activate NF-κB *via* TRAF-6 recruitment (86) and induce the expression of the chemotactic factor CCL22, which hampers migration of CCR4-expressing NK cells (87). Regarding UL140, we assume that it has been affected together with the other genes mainly due to its adjacent location in the highly variable UL/b′ region.

This scenario becomes even more complicated when the contribution of multiple-strain infection (i.e., mixed infection, super-infection) is taken into account (34, 88–90). According to recent data, mixed infection (31, 34) and extensive recombination (30, 34, 91) of genetically distinct strains enhance HCMV genetic variability. The occurrence of multiple-strain infection seems, however, an unlikely event in our case given that NGS analysis showed all our five HCMV clinical strains are derived from single-strain infections.

Our data also show clustering of high-frequency variants in specific regions corresponding to the *UL20*-*UL24*, *UL48*, and *UL84-UL87* genes in the “aggressive” strains P14 and P15 but not in the “moderate” strain P4 (**Supplementary Figure 3**), probably due to an ongoing adaptation of these strains to cell culture conditions. If this was indeed the case, it would be quite surprising that, despite sharing similar genetic background, only the “aggressive” P14 and P15 strains but not the “moderate” P4 show signs of cell culture adaptation. The reasons for this observation are presently unclear. An additional level of complexity derives from the fact that the association between variability within the *UL20*, *UL22A*, *UL24*, and *UL37* gene regions and cell culture adaptation is highly controversial. While a global mutagenesis approach has shown that UL20 is dispensable for HCMV growth in primary human fibroblasts, two large-scale mutagenic analyses showed that mutations in overlapping UL21a/UL21 resulted in a severe defect of virus growth in fibroblasts (92). Furthermore, an early sequence inspection of UL20 revealed a distant homology to the TCR-γ chain, implying a possible role in promoting viral infection or immune evasion (93). UL23, another polymorphic component of this cluster, has been shown to inhibit STAT1-dependent transcription of IFN-γ stimulated genes by binding to human N-myc interactor protein (Nmi) and blocking its association with STAT1 (94). Thus, it is tempting to speculate that the genetic variability of UL23 may additionally contribute to the functional features of the “aggressive” strains.

Another important finding of our study is the identification of different variants of the *UL48* gene, critical for viral replication, in P14 and P15. Indeed, the N-terminal region of pUL48 displays a deubiquitinase activity, which moderately promotes viral growth in cultured fibroblasts (95, 96), and a nuclear localization signal (NLS) is required for viral growth (97). Moreover, pUL48 directs pUL47 to the viral assembly complex (vAC) to promote tegumentation and maturation of viral capsids (98). Given that pUL48 is the largest HCMV protein with multiple functions, the “aggressive” strains may have evolved UL48 variants to enhance their replication.

The strength of our study includes the comprehensive analysis of the entire genome of several HCMV clinical strains by NGS, an approach that has allowed us to uncover the loss of multiple immunomodulatory genes and detect single variants in our clinical isolates. This is particularly important considering the scarce literature addressing the relationship between HCMV genetic variability and immune modulation.

The limitations of this study include the passaging of HCMV clinical isolates, albeit kept to a minimum, to amplify the virus from initially low-titered samples and the *in vitro* immunological assays, which could result in inevitable culture adaptation and selection of mutants. Nevertheless, the need to study viral pathogenicity requires the use of clinical strains as a model closest to that resembling *in vivo* infection, which may shed light on viral genome-host interactions and immune selective pressure. Moreover, we note that the possibility of multiple-strain infections occurring in a single patient should be further addressed.

Altogether, our findings show for the first time that newly obtained clinical HCMV isolates, in addition to their existing genetic heterogeneity, are able to translate these differences into antiviral effector functions. Although further studies based on a higher number of patients should be greatly encouraged to reach a statistical significance, our study suggests that viral determinants are genetically and functionally different from patient to patient. Therefore, the ability of distinct HCMV clinical isolates to trigger different immune responses should be considered when designing vaccines or developing a more personalized treatment for HCMV disease.

## Data Availability Statement

The raw data supporting the conclusions of this manuscript will be made available by the authors, without undue reservation, to any qualified researcher.

## Ethics Statement

The studies involving human participants were reviewed and approved by A.O.U. Città della Salute e della Scienza di Torino – A.O. Ordine Mauriziano – A.S.L. TO1” (No. 007816). Written informed consent to participate in this study was provided by the participants’ legal guardian/next of kin.

## Author Contributions

Study design: CC, VD, GG, and MS. Laboratory analyses: GG, SM, CC, MB, and LS. Patients’ management: AC, EB, and AL. Statistical/phylogenetic analyses: MS, DF, TS, LS, and RC. Manuscript writing: CC, VD, GG, and MS. Critical revision of the manuscript: MA, SL, and AS. All authors contributed to the article and approved the submitted version.

## Funding

This work was supported by the European Commission under the Horizon2020 program (H2020-MSCA-ITN-2015), Italian Ministry of Education, University and Research-MIUR (PRIN 2015 to MA and CC, 2015W729WH; PRIN 2015 to VD, 2015RMNSTA; PRIN 2017 to VD and CC, 2017 20178ALPCM), and Italian Ministry of Health (grant No. RC 2019 to MS, grant No. RC 2018-2019 to DF). The funding agencies had no role in study design, data collection and interpretation, as well as in the decision to submit this work for publication.

## Conflict of Interest

The authors declare that the research was conducted in the absence of any commercial or financial relationships that could be construed as a potential conflict of interest.
