# Supplementary material for: Genetic Variability of Human Cytomegalovirus Clinical Isolates Correlates With Altered Expression of Natural Killer Cell-Activating Ligands and IFN-γ
Source: Front Immunol. 2021 Apr 9;12:532484. doi: 10.3389/fimmu.2021.532484 (PMC8062705; doi:10.3389/fimmu.2021.532484)
Supplement: Supplementary file 1 [file Image_1.pdf]

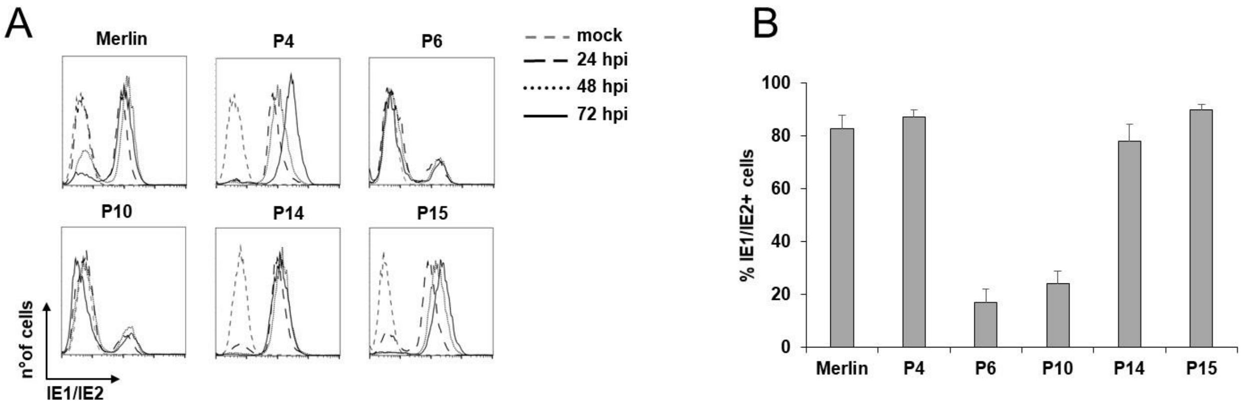

	24 hpi	48 hpi	72 hpi
**Merlin**	44	70	85
**P4**	92	96	94
**P6**	19	21	20
**P10**	15	21	18
**P14**	86	96	96
**P15**	77	95	94

Supplementary Figure 1. Analysis of the percentage of IE1/IE2 positive (+) cells in HFFs infected with HCMV clinical isolates. Primary human foreskin fibroblasts (HFFs) infected with the indicated clinical isolates (P4, 6, 10, 14, and 15), the Merlin strain, or left uninfected (mock) were co-cultured with an excess of uninfected HFFs, as described in Materials and Methods. The percentage of IE+ cells was analysed by FACS after intracellular staining with a specific anti-IE1/IE2 mAb. **(A)** *Top panels*, FACS plots from one representative experiment performed at the indicated days post-infection; *bottom panel*, numbers indicate the percentage of IE1/IE2+ cells. **(B)** The percentage of IE1/IE2+ cells is expressed as mean ± SE from at least six independent experiments performed at 3 dpi.
