# Supplementary material for: Genetic Variability of Human Cytomegalovirus Clinical Isolates Correlates With Altered Expression of Natural Killer Cell-Activating Ligands and IFN-γ
Source: Front Immunol. 2021 Apr 9;12:532484. doi: 10.3389/fimmu.2021.532484 (PMC8062705; doi:10.3389/fimmu.2021.532484)
Supplement: Supplementary file 2 [file Image_2.pdf]

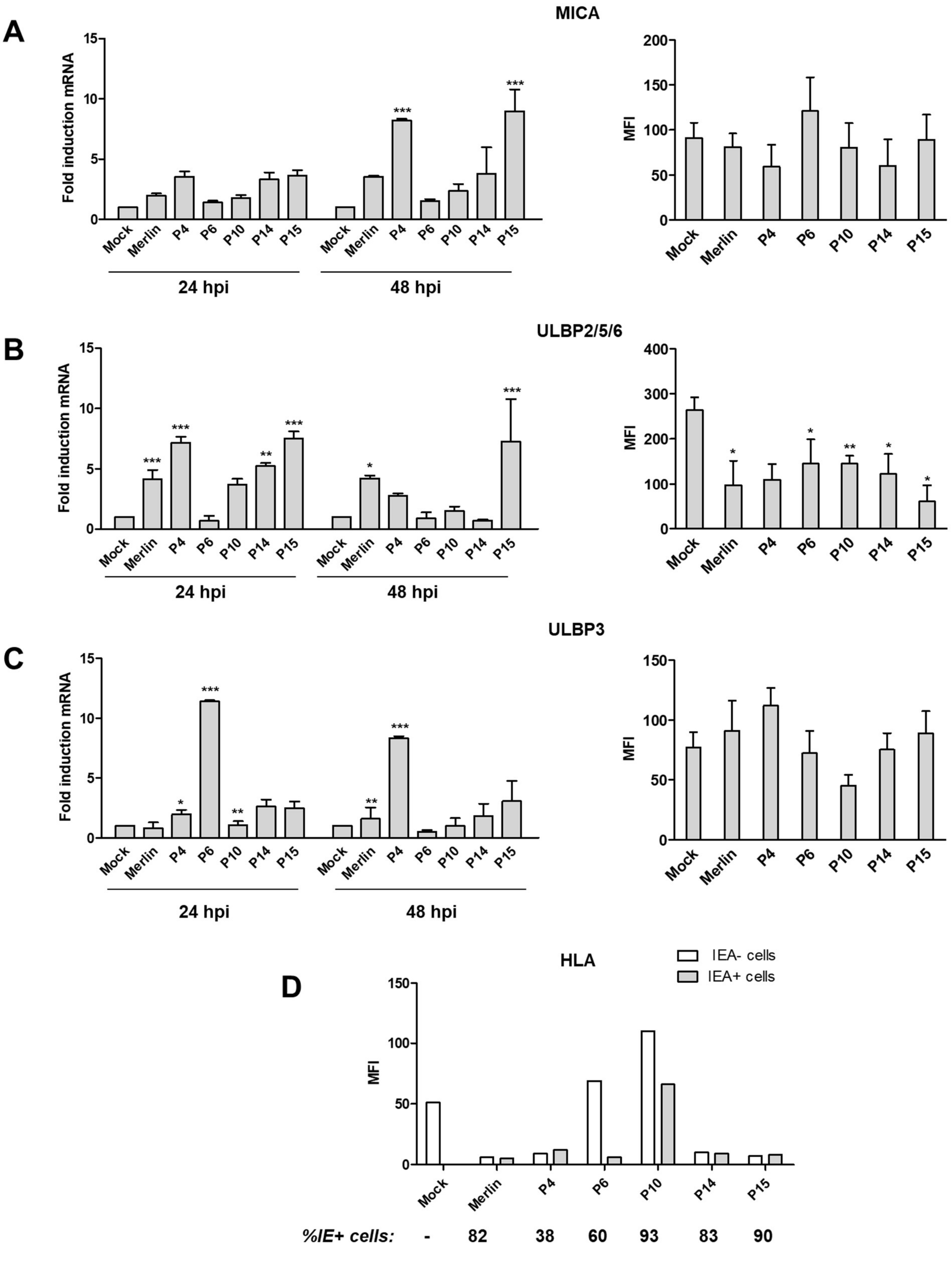

A
MICA
Fold induction mRNA
Mock
Merlin
P4
P6
P10
P14
P15
24 hpi
48 hpi
MFI
B
ULBP2/5/6
C
ULBP3
D
HLA
IEA- cells
IEA+ cells
%IE+ cells:
-
82
38
60
93
83
90

Supplementary Figure 2. NKG2D ligand expression upon infection of HFFs with different HCMV isolates. Cells were infected with the indicated HCMV isolates (P), the Merlin strain, or uninfected (mock), as described in the legend to Figure 1. **(A)** MICA. **(B)** ULBP2/5/6. **(C)** ULBP3. *Lefts panels:* ligand expression was evaluated at the mRNA level by RT-qPCR at 24 and 48 hours post infection (hpi) Values were normalized to the housekeeping gene glyceraldehyde-3-phosphate dehydrogenase (GAPDH) mRNA and plotted as fold induction relative to mock-infected cells (set as 1). Data from three experiments performed at 24 and 48 hours post-infection (hpi) are shown. Error bars show standard deviation (SD) (*$P < 0.05$; **$P < 0.01$; ***$P < 0.001$; two-way ANOVA followed by Bonferroni's post-tests, for comparison of infected *vs.* mock cells).
Right panels: FACS analysis at 3 days post infection (dpi). Data are from at least two (MICA) or three (ULBP2/5/6 and ULBP3) experiments performed with all HCMV isolates. Expression levels are expressed as MFI ± SD (*$P < 0.05$; **$P < 0.01$ paired Student t test for comparison of infected *vs.* mock cells). (**D**) Modulation of HLA-I expression by HCMV clinical isolates. HFFs infected with the indicated clinical isolates (P4, 6, 10, 14, and 15), the Merlin strain, or uninfected (mock) were co-cultured with an excess of uninfected HFFs and subjected to FACS analysis at 3 days post-infection. Cell surface HLA-I expression levels were determined in cells positive or negative for HCMV IE1/IE2, after intracellular staining with a specific anti-IE1/IE2 mAb. In the bottom part, numbers indicate the percentage of IE1/IE2+ cells at 3 days post-infection.
