# Supplementary material for: Genetic Variability of Human Cytomegalovirus Clinical Isolates Correlates With Altered Expression of Natural Killer Cell-Activating Ligands and IFN-γ
Source: Front Immunol. 2021 Apr 9;12:532484. doi: 10.3389/fimmu.2021.532484 (PMC8062705; doi:10.3389/fimmu.2021.532484)
Supplement: Supplementary file 3 [file Image_3.pdf]

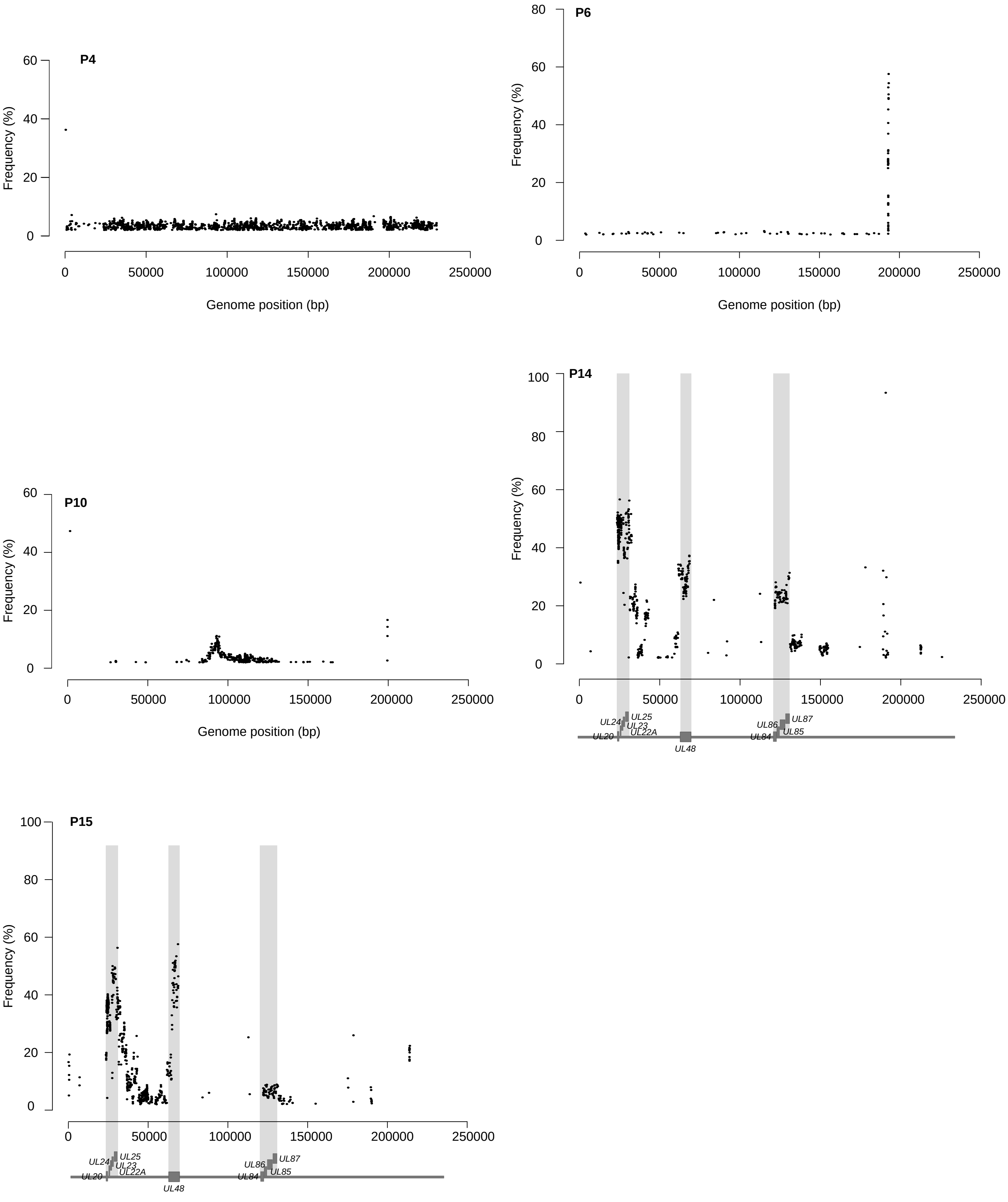

Supplementary Figure 3. Distribution of heterozygous variants. The genomic location and frequency of heterozygous variants is shown for all isolates. For P14 and P15, a schematic representation of the HCMV genome is shown at the bottom to visualize the regions where most high-frequency variants occur (shaded areas).
