# Supplementary material for: Genetic Variability of Human Cytomegalovirus Clinical Isolates Correlates With Altered Expression of Natural Killer Cell-Activating Ligands and IFN-γ
Source: Front Immunol. 2021 Apr 9;12:532484. doi: 10.3389/fimmu.2021.532484 (PMC8062705; doi:10.3389/fimmu.2021.532484)
Supplement: Supplementary file 4 [file Image_4.pdf]

Supplementary Figure 4. Protein alignment of proteins involved in the modulation of NK cell activation. The alignments of known proteins involved in NK cell modulation are shown for the five isolates. Amino acid changes are marked in red.

RL11

```
P4_RL11        MQTYSTPLTLVIVTSLFLFTTQGSSSNAVEPTKKPLKLANYRATCEDRTRTLVTRLNTSH
P6_RL11        MQTYSTPLTLVIVTSLFLFTTQGSSSNAVEPTKKPLKLANYRATCEDRTRTLVTRLNTSH
P14_RL11       MQTYSTPLTLVIVTSLFLFTTQGSSSNAVEPTKKPLKLANYRATCEDRTRTLVTRLNTSH
P15_RL11       MQTYSTPLTLVIVTSLFLFTTQGSSSNAVEPTKKPLKLANYRATCEDRTRTLVTRLNTSH
P10_RL11       MQTYSTPLTLAIVTSLFLFTTQGGSSNAVEPTKKPLKLANYRATCEDRTRTLVTRLNTSH

P4_RL11        HSVVWQRYDIYSRYMRRMPPLCIITDAYKETTHQGGATFTCTRQNLTLYNLTVKDTGVYL
P6_RL11        HSVVWQRYDIYSRYMRRMPPLCIITDAYKETTHQGGATFTCTRQNLTLYNLTVKDTGVYL
P14_RL11       HSVVWQRYDIYSRYMRRMPPLCIITDAYKETTHQGGATFTCTRQNLTLYNLTVKDTGVYL
P15_RL11       HSVVWQRYDIYSRYMRRMPPLCIITDAYKETTHQGGATFTCTRQNLTLYNLTVKDTGVYL
P10_RL11       HSVVWQRYDIYSRYMRRMPPLCIITDAYKETTHQGGATFTCTRQNLTLYNLTVKDTGVYL

P4_RL11        LQDQYTGDVEAFYLIIHPRSFCRALETRRCFYPGPGRVVVTDSQEADRAIISDLKRQWSG
P6_RL11        LQDQYTGDVEAFYLIIHPRSFCRALETRRCFYPGPGRVVVTDSQEADRAIISDLKRQWSG
P14_RL11       LQDQYTGDVEAFYLIIHPRSFCRALETRRCFYPGPGRVVVTDSQEADRAIISDLKRQWSG
P15_RL11       LQDQYTGDVEAFYLIIHPRSFCRALETRRCFYPGPGRVVVTDSQEADRAIISDLKRQWSG
P10_RL11       LQDQYTGDVEAFYLIIHPRSFCRALETRRCFYPGPGRVVVTDSQEADRAIISDLKRQWSG

P4_RL11        LSLHCAWVSGLMIFVGALVICFLRSQRIGEQDAERLRTDLDTEPLLLTVDGDLE
P6_RL11        LSLHCAWVSGLMIFVGALVICFLRSQRIGEQDAERLRTDLDTEPLLLTVDGDLE
P14_RL11       LSLHCAWVSGLMIFVGALVICFLRSQRIGEQDAERLRTDLDTEPLLLTVDGDLE
P15_RL11       LSLHCAWVSGLMIFVGALVICFLRSQRIGEQDAERLRTDLDTEPLLLTVDGDLE
P10_RL11       LSLHCAWVSGLMIFVGALVICFLRSQRIGEQDAEQLRTDLDTEPLLLTVDGDLE
```

UL16

```
P4_UL16        MERRRGTVPLGWVFFVLCLSASSPCAVDLGSKSSNSTCRLNVTELASIHPGETWTLHGMC
P6_UL16        MERRRGTVPLGWVFFVLCLSASSPCAVDLGSKSSNSTCRLNVTELASIHPGETWTLHGMC
P14_UL16       MERRRGTVPLGWVFFVLCLSASSPCAVDLGSKSSNSTCRLNVTELASIHPGETWTLHGMC
P15_UL16       MERRRGTVPLGWVFFVLCLSASSPCAVDLGSKSSNSTCRLNVTELASIHPGETWTLHGMC
P10_UL16       MERRRGTVPLGWVFFVLCLSASSSCAVDLGSKSSNSTCRLNVTELASIHPGETWTLHGMC

P4_UL16        ISICYYENVTEDEIIGVAFTWQHNESVVDLWLYQNDTVIRNFSDITTNILQDGLKMRTVP
P6_UL16        ISICYYENVTEDEIIGVAFTWQHNESVVDLWLYQNDTVIRNFSDITTNILQDGLKMRTVP
P14_UL16       ISICYYENVTEDEIIGVAFTWQHNESVVDLWLYQNDTVIRNFSDITTNILQDGLKMRTVP
P15_UL16       ISICYYENVTEDEIIGVAFTWQHNESVVDLWLYQNDTVIRNFSDITTNILQDGLKMRTVP
P10_UL16       ISICYYENVTEDEIIGVAFTWQHNESVVDLWLYQNDTVIRNFSDITTNILQDGLKMRTVP

P4_UL16        VTKLYTSRMVTNLTVGRYDCLRCENGTMKIIERLYVRLGSLYPRPPGSGLAKHPSVSADE
P6_UL16        VTKLYTSRMVTNLTVGRYDCLRCENGTMKIIERLYVRLGSLYPRPPGSGLAKHPSVSADE
P14_UL16       VTKLYTSRMVTNLTVGRYDCLRCENGTMKIIERLYVRLGSLYPRPPGSGLAKHPSVSADE
P15_UL16       VTKLYTSRMVTNLTVGRYDCLRCENGTMKIIERLYVRLGSLYPRPPGSGLAKHPSVSADE
P10_UL16       VTKLYTSRMVTNLTVGRYDCLRCENGTTKIIERLYVRLGSLYPRPPGSGLAKHPSVSADE

P4_UL16        ELSATLARDIVLVSAITLFFFLLALRIPQRLCQRLRIRLPHRYQRLRTED
P6_UL16        ELSATLARDIVLVSAITLFFFLLALRIPQRLCQRLRIRLPHRYQRLRTED
P14_UL16       ELSATLARDIVLVSAITLFFFLLALRIPQRLCQRLRIRLPHRYQRLRTED
P15_UL16       ELSATLARDIVLVSAITLFFFLLALRIPQRLCQRLRIRLPHRYQRLRTED
P10_UL16       ELSATLARDIVLVSAITLFFFLLALRIPQRLCQRLRIRLPHRYQRLRTED
```

UL18

```
P4_UL18        MMTTWCLTLFVLWMLRVVGMHVLRYGYTGIFDDTSHMTLTVVGIFDGQHFFTYHVNSSDR
P6_UL18        MMTTWCLTLFVLWMLRVVGMHVLRYGYTGIFDDTSHMTLTVVGIFDGQHFFTYHVNSSDR
P14_UL18       MMTTWCLTLFVLWMLRVVGMHVLRYGYTGIFDDTSHMTLTVVGIFDGQHFFTYHVNSSDR
P15_UL18       MMTTWCLTLFVLWMLRVVGMHVLRYGYTGIFDDTSHMTLTVVGIFDGQHFFTYHVNSSDR
P10_UL18       MMTMWCLTLFVLWMLRVVGMHVLRYGYTGIFDDTSHMTLTVVGIFDGQHFFTYHVNSSDK

P4_UL18        TSSRANGTISWMANVSAAYPTYLDGERAKGDLIFNQTEQNLLELEIALGYRSQSVLTWTH
P6_UL18        TSSRANGTISWMANVSAAYPTYLDGERAKGDLIFNQTEQNLLELEIALGYRSQSVLTWTH
P14_UL18       TSSRANGTISWMANVSAAYPTYLDGERAKGDLIFNQTEQNLLELEIALGYRSQSVLTWTH
```

```
P15_UL18        TSSRANGTISWMANVSAAYPTYLDGERAKGDLIFNQTEQNLLELEIALGYRSQSVLTWTH
P10_UL18        ASSRANGTISWMANVSAAYPTYLDGERAKGDLIFNQTEQNLLELEIALGYRSQSVLTWTH

P4_UL18         ECNTTENGSFVAGYEGFGWDGETLMELKDNLTLWTGPNYEISWLKQNKTYIDGKIKNISE
P6_UL18         ECNTTENGSFVAGYEGFGWDGETLMELKDNLTLWTGPNYEISWLKQNKTYIDGKIKNISE
P14_UL18        ECNTTENGSFVAGYEGFGWDGETLMELKDNLTLWTGPNYEISWLKQNKTYIDGKIKNISE
P15_UL18        ECNTTENGSFVAGYEGFGWDGETLMELKDNLTLWTGPNYEISWLKQNKTYIDGKIKNISE
P10_UL18        ECNTTENGSFVAGYEGFGWDGETLMELKDNLTLWTGPNYEISWLKQNKTYIDGKIKNISE

P4_UL18         GDTTIQRNYLKGNCTQWSVIYSGFQTPVTHPVVKGGVRNQNDNRAEAFCTSYGFFPGEIN
P6_UL18         GDTTIQRNYLKGNCTQWSVIYSGFQTPVTHPVVKGGVRNQNDNRAEAFCTSYGFFPGEIN
P14_UL18        GDTTIQRNYLKGNCTQWSVIYSGFQTPVTHPVVKGGVRNQNDNRAEAFCTSYGFFPGEIN
P15_UL18        GDTTIQRNYLKGNCTQWSVIYSGFQTPVTHPVVKGGVRNQNDNRAEAFCTSYGFFPGEIN
P10_UL18        GDTTIQRNYLKGNCTQWSVIYSGFQTPVTHPVVKGGVRNQNDNRAEAFCTSYGFFPGEIN

P4_UL18         ITFIHYGDKVPEDSEPQCNPLLPTLDGTFHQGCYVAIFCNQNYTCRVTHGNWTVEIPISV
P6_UL18         ITFIHYGDKVPEDSEPQCNPLLPTLDGTFHQGCYVAIFCNQNYTCRVTHGNWTVEIPISV
P14_UL18        ITFIHYGDKVPEDSEPQCNPLLPTLDGTFHQGCYVAIFCNQNYTCRVTHGNWTVEIPISV
P15_UL18        ITFIHYGDKVPEDSEPQCNPLLPTLDGTFHQGCYVAIFCNQNYTCRVTHGNWTVEIPISV
P10_UL18        ITFIHYGNKAPDDSEPQCNPLLPTLDGTFHQGCYVAIFCNQNYTCRVTHGNWTVEIPISV

P4_UL18         TSPDDSSSGEVPDHPTANKRYNTMTISSVLLALLLCALLFAFLHYFTTLKQYLRNLAFAW
P6_UL18         TSPDDSSSGEVPDHPTANKRYNTMTISSVLLALLLCALLFAFLHYFTTLKQYLRNLAFAW
P14_UL18        TSPDDSSSGEVPDHPTANKRYNTMTISSVLLALLLCALLFAFLHYFTTLKQYLRNLAFAW
P15_UL18        TSPDDSSSGEVPDHPTANKRYNTMTISSVLLALLLCALLFAFLHYFTTLKQYLRNLAFAW
P10_UL18        TSPDDSSSGEVPDHPTANKRYNTMTISSVLLALLLCALLFAFLHYFTTLKQYLRNLAFAW

P4_UL18         RYRKVRSS
P6_UL18         RYRKVRSS
P14_UL18        RYRKVRSS
P15_UL18        RYRKVRSS
P10_UL18        RYRKVRSS
```

UL40

```
P4_UL40         MNKFSNTRIGFTCAVMAPRTLVLTLGLLCMRIRSLLSSPAETTVTTAGVTSAHGPLCPLV
P6_UL40         MNKFSNTRIGFTCAVMAPRTLVLTLGLLCMRIRSLLSSPAETTVTTAGVTSAHGPLCPLV
P14_UL40        MNKFSNTRIGFTCAVMAPRTLVLTLGLLCMRIRSLLSSPAETTVTTAGVTSAHGPLCPLV
P15_UL40        MNKFSNTRIGFTCAVMAPRTLVLTLGLLCMRIRSLLSSPAETTVTTAGVTSAHGPLCPLV
P10_UL40        MNKFSNTRIGFTCAVVAPRTLILTLGLLCMRIRSLLSSPAETTVTTAGVTSAHGPLCPLV

P4_UL40         FQGWAYAVYHQGDMALMTLDVYCCRQTSNNTAVAFSRHLAVNTLLIEVGNNTHRRADGVS
P6_UL40         FQGWAYAVYHQGDMALMTLDVYCCRQTSNNTAVAFSRHLAVNTLLIEVGNNTHRRADGVS
P14_UL40        FQGWAYAVYHQGDMALMTLDVYCCRQTSNNTAVAFSRHLAVNTLLIEVGNNTHRRADGVS
P15_UL40        FQGWAYAVYHQGDMALMTLDVYCCRQTSNNTAVAFSRHLAVNTLLIEVGNNTHRRADGVS
P10_UL40        FQGWAYAVYHQGDMALMTLDVYCCRQTSNNTAVAFSRHLAVNTLLIEVGNNTRRRADGVS

P4_UL40         CLDHFRAQHQDCPAQTVHVRGVNESAFGITHLQSCCLNEHSQLSERVAYHLKLRPATFGL
P6_UL40         CLDHFRAQHQDCPAQTVHVRGVNESAFGITHLQSCCLNEHSQLSERVAYHLKLRPATFGL
P14_UL40        CLDHFRAQHQDCPAQTVHVRGVNESAFGITHLQSCCLNEHSQLSERVAYHLKLRPATFGL
P15_UL40        CLDHFRAQHQDCPAQTVHVRGVNESAFGITHLQSCCLNEHSQLSERVAYHLKLRPATFGL
P10_UL40        CLDHFRAQHQDCPAQTVHVRGVNESAFGLTHLQSCCLNEHSQLSERVAYHLKLRPATFGL

P4_UL40         ETWAMYTVGILALGSFSSFYSQIARSLGVLPNDHHYALKKA
P6_UL40         ETWAMYTVGILALGSFSSFYSQIARSLGVLPNDHHYALKKA
P14_UL40        ETWAMYTVGILALGSFSSFYSQIARSLGVLPNDHHYALKKA
P15_UL40        ETWAMYTVGILALGSFSSFYSQIARSLGVLPNDHHYALKKA
P10_UL40        ETWAMYTVGILALGSFSSFYSQIARSLGVLPNDHHYALKKA
```

UL83

```
P4_UL83         MESRGRRCPDMISVLGPISGHVLKAVFSRGDTPVLPHETRLLQTGIHVRVSQPSLILVSQ
P6_UL83         MESRGRRCPDMISVLGPISGHVLKAVFSRGDTPVLPHETRLLQTGIHVRVSQPSLILVSQ
P14_UL83        MESRGRRCPDMISVLGPISGHVLKAVFSRGDTPVLPHETRLLQTGIHVRVSQPSLILVSQ
P15_UL83        MESRGRRCPDMISVLGPISGHVLKAVFSRGDTPVLPHETRLLQTGIHVRVSQPSLILVSQ
P10_UL83        MESRGRRCPEMISVLGPISGHVLKAVFSRGDTPVLPHETRLLQTGIHVRVSQPSLILVSQ

P4_UL83         YTPDSTPCHRGDNQLQVQHTYFTGSEVENVSVNVHNPTGRSICPSQEPMSIYVYALPLKM
P6_UL83         YTPDSTPCHRGDNQLQVQHTYFTGSEVENVSVNVHNPTGRSICPSQEPMSIYVYALPLKM
P14_UL83        YTPDSTPCHRGDNQLQVQHTYFTGSEVENVSVNVHNPTGRSICPSQEPMSIYVYALPLKM
```

```
P15_UL83        YTPDSTPCHRGDNQLQVQHTYFTGSEVENVSVNVHNPTGRSICPSQEPMSIYVYALPLKM
P10_UL83        YTPDSTPCHRGDNQLQVQHTYFTGSEVENVSVNVHNPTGRSICPSQEPMSIYVYALPLKM

P4_UL83         LNIPSINVHHYPSAAERKHRHLPVADAVIHASGKQMWQARLTVSGLAWTRQQNQWKEPDV
P6_UL83         LNIPSINVHHYPSAAERKHRHLPVADAVIHASGKQMWQARLTVSGLAWTRQQNQWKEPDV
P14_UL83        LNIPSINVHHYPSAAERKHRHLPVADAVIHASGKQMWQARLTVSGLAWTRQQNQWKEPDV
P15_UL83        LNIPSINVHHYPSAAERKHRHLPVADAVIHASGKQMWQARLTVSGLAWTRQQNQWKEPDV
P10_UL83        LNIPSINVHHYPSAAERKHRHLPVADAVIHASGKQMWQARLTVSGLAWTRQQNQWKEPDV

P4_UL83         YYTSAFVFPTKDVALRHVVCAHELVCSMENTRATKMQVIGDQYVKVYLESFCEDVPSGKL
P6_UL83         YYTSAFVFPTKDVALRHVVCAHELVCSMENTRATKMQVIGDQYVKVYLESFCEDVPSGKL
P14_UL83        YYTSAFVFPTKDVALRHVVCAHELVCSMENTRATKMQVIGDQYVKVYLESFCEDVPSGKL
P15_UL83        YYTSAFVFPTKDVALRHVVCAHELVCSMENTRATKMQVIGDQYVKVYLESFCEDVPSGKL
P10_UL83        YYTSAFVFPTKDVALRHVVCAHELVCSMENTRATKMQVIGDQYVKVYLESFCEDVPSGKL

P4_UL83         FMHVTLGSDVEEDLTMTRNPQPFMRPHERNGFTVLCPKNMIIKPGKISHIMLDVAFTSHE
P6_UL83         FMHVTLGSDVEEDLTMTRNPQPFMRPHERNGFTVLCPKNMIIKPGKISHIMLDVAFTSHE
P14_UL83        FMHVTLGSDVEEDLTMTRNPQPFMRPHERNGFTVLCPKNMIIKPGKISHIMLDVAFTSHE
P15_UL83        FMHVTLGSDVEEDLTMTRNPQPFMRPHERNGFTVLCPKNMIIKPGKISHIMLDVAFTSHE
P10_UL83        FMHVTLGSDVEEDLTMTRNPQPFMRPHERNGFTVLCPKNMIIKPGKISHIMLDVAFTSHE

P4_UL83         HFGLLCPKSIPGLSISGNLLMNGQQIFLEVQAIRETVELRQYDPVAALFFFDIDLLLQRG
P6_UL83         HFGLLCPKSIPGLSISGNLLMNGQQIFLEVQAIRETVELRQYDPVAALFFFDIDLLLQRG
P14_UL83        HFGLLCPKSIPGLSISGNLLMNGQQIFLEVQAIRETVELRQYDPVAALFFFDIDLLLQRG
P15_UL83        HFGLLCPKSIPGLSISGNLLMNGQQIFLEVQAIRETVELRQYDPVAALFFFDIDLLLQRG
P10_UL83        HFGLLCPKSIPGLSISGNLLMNGQQIFLEVQAIRETVELRQYDPVAALFFFDIDLLLQRG

P4_UL83         PQYSEHPTFTSQYRIQGKLEYRHTWDRHDEGAAQGDDDVWTSGSDSDEELVTTERKTPRV
P6_UL83         PQYSEHPTFTSQYRIQGKLEYRHTWDRHDEGAAQGDDDVWTSGSDSDEELVTTERKTPRV
P14_UL83        PQYSEHPTFTSQYRIQGKLEYRHTWDRHDEGAAQGDDDVWTSGSDSDEELVTTERKTPRV
P15_UL83        PQYSEHPTFTSQYRIQGKLEYRHTWDRHDEGAAQGDDDVWTSGSDSDEELVTTERKTPRV
P10_UL83        PQYSEHPTFTSQYRIQGKLEYRHTWDRHDEGAAQGDDDVWTSGSDSDEELVTTERKTPRV

P4_UL83         TGGGAMAGASTSAGRKRKSASSATACTAGVMTRGRLKAESTVAPEEDTDEDSDNEIHNPA
P6_UL83         TGGGAMAGASTSAGRKRKSASSATACTAGVMTRGRLKAESTVAPEEDTDEDSDNEIHNPA
P14_UL83        TGGGAMAGASTSAGRKRKSASSATACTAGVMTRGRLKAESTVAPEEDTDEDSDNEIHNPA
P15_UL83        TGGGAMAGASTSAGRKRKSASSATACTAGVMTRGRLKAESTVAPEEDTDEDSDNEIHNPA
P10_UL83        TGGGAMAGASTSAGRKRKSASSATACTSGVMTRGRLKAESTVAPEEDTDEDSDNEIHNPA

P4_UL83         VFTWPPWQAGILARNLVPMVATVQGQNLKYQEFFWDANDIYRIFAELEGVWQPAAQPKRR
P6_UL83         VFTWPPWQAGILARNLVPMVATVQGQNLKYQEFFWDANDIYRIFAELEGVWQPAAQPKRR
P14_UL83        VFTWPPWQAGILARNLVPMVATVQGQNLKYQEFFWDANDIYRIFAELEGVWQPAAQPKRR
P15_UL83        VFTWPPWQAGILARNLVPMVATVQGQNLKYQEFFWDANDIYRIFAELEGVWQPAAQPKRR
P10_UL83        VFTWPPWQAGILARNLVPMVATVQGQNLKYQEFFWDANDIYRIFAELEGVWQPAAQPKRR

P4_UL83         RHRQEALPGPCIASTPKKHRG
P6_UL83         RHRQEALPGPCIASTPKKHRG
P14_UL83        RHRQEALPGPCIASTPKKHRG
P15_UL83        RHRQEALPGPCIASTPKKHRG
P10_UL83        RHRQEALPGPCIASTPKKHRG
```

UL112

```
P4_UL112        MDLPTTVVRKYWTFANPNRILHQSVNQTFDVRQFVFDTARLVNCVDGDGKVLHLNKGWLC
P6_UL112        MDLPTTVVRKYWTFANPNRILHQSVNQTFDVRQFVFDTARLVNCVDGDGKVLHLNKGWLC
P14_UL112       MDLPTTVVRKYWTFANPNRILHQSVNQTFDVRQFVFDTARLVNCVDGDGKVLHLNKGWLC
P15_UL112       MDLPTTVVRKYWTFANPNRILHQSVNQTFDVRQFVFDTARLVNCVDGDGKVLHLNKGWLC
P10_UL112       MDLPTTVVRKYWTFANPNRILHQSVNQTFDVRQFVFDTARLVNCVDGDGKVLHLNKGWLC

P4_UL112        ATIMQHGEASAGAKTQQGFMSIDITGDGELQEHLFVRGGIVFNKSVSSVVGSSGPNESAL
P6_UL112        ATIMQHGEASAGAKTQQGFMSIDITGDGELQEHLFVRGGIVFNKSVSSVVGSSGPNESAL
P14_UL112       ATIMQHGEASAGAKTQQGFMSIDITGDGELQEHLFVRGGIVFNKSVSSVVGSSGPNESAL
P15_UL112       ATIMQHGEASAGAKTQQGFMSIDITGDGELQEHLFVRGGIVFNKSVSSVVGSSGPNESAL
P10_UL112       ATIMQHGEASAGAKTQQGFMSIDITGDGELQEHLFVRGGIVFNKSVSSVVGSSGPNESAL

P4_UL112        LTMISENGNLQVTYVRHYLKNHGESSSGGGGCGAASTASAVCVSSLGGSGGTRDGPSAEE
P6_UL112        LTMISENGNLQVTYVRHYLKNHGESSSGGGGCGAASTASAVCVSSLGGSGGTRDGPSAEE
P14_UL112       LTMISENGNLQVTYVRHYLKNHGESSSGGGGCGAASTASAVCVSSLGGSGGTRDGPSAEE
P15_UL112       LTMISENGNLQVTYVRHYLKNHGESSSGGGGCGAASTASAVCVSSLGGSGGTRDGPSAEE
P10_UL112       LTMISENGNLQVTYVRHYLKNHGESSGGGGGCGAASTASAVCVSSLGGSGGTRDGPSAEE

P4_UL112        QQRRRQEQRHEERRKKSSSSAGGGGGGGTGGGGGGGGSGGQHSSDSANGLLRDPRLMNRQ
```

P6_UL112 QQRRRQEQRHEERRKKSSSSAGGGGGGGTGGGGGGGGSGGQHSSDSANGLLRDPRLMNRQ
P14_UL112 QQRRRQEQRHEERRKKSSSSAGGGGGGGTGGGGGGGGSGGQHSSDSANGLLRDPRLMNRQ
P15_UL112 QQRRRQEQRHEERRKKSSSSAGGGGGGGTGGGGGGGGSGGQHSSDSANGLLRDPRLMNRQ
P10_UL112 QQRRRQEQRHEERRKKSSSSAGGGGGGGAGGGGGGGGSGGQHSSDSANGLLRDPRLMNRQ

P4_UL112 KERRPPPSSENDGSPPLREAKRQKTTAQHEGHGGGGKNETEQQSGGAGGGGGGGSGRMSL
P6_UL112 KERRPPPSSENDGSPPLREAKRQKTTAQHEGHGGGGKNETEQQSGGAGGGGGGGSGRMSL
P14_UL112 KERRPPPSSENDGSPPLREAKRQKTTAQHEGHGGGGKNETEQQSGGAGGGGGGGSGRMSL
P15_UL112 KERRPPPSSENDGSPPLREAKRQKTTAQHEGHGGGGKNETEQQSGGAGGGGGGGSGRMSL
P10_UL112 KERRPPPSSENDGSPPLREAKRQKTTAQHEGHGGGGKNETEQQSGGAGGGGGGGSGRMSL

P4_UL112 PLDTSEAVAFLNYSSSSSAVSSSSNNHHHHHHHHNAVTDVAAGTDGALLLPIERGAVVSS
P6_UL112 PLDTSEAVAFLNYSSSSSAVSSSSNNHHHHHHHHNAVTDVAAGTDGALLLPIERGAVVSS
P14_UL112 PLDTSEAVAFLNYSSSSSAVSSSSNNHHHHHHHHNAVTDVAAGTDGALLLPIERGAVVSS
P15_UL112 PLDTSEAVAFLNYSSSSSAVSSSSNNHHHHHHHHNAVTDVAAGTDGALLLPIERGAVVSS
P10_UL112 PLDTSEAVAFLNYSSSSSAVSSSSNNHHHHHHHHNAVTDVAAGTDGALLLPIERGAVVSS

P4_UL112 PSSTSPSSLLSLPRPSSAHSAGETVQESEAAATAAAAGLMMMRRMRRAPAEAAEAPPQSE
P6_UL112 PSSTSPSSLLSLPRPSSAHSAGETVQESEAAATAAAAGLMMMRRMRRAPAEAAEAPPQSE
P14_UL112 PSSTSPSSLLSLPRPSSAHSAGETVQESEAAATAAAAGLMMMRRMRRAPAEAAEAPPQSE
P15_UL112 PSSTSPSSLLSLPRPSSAHSAGETVQESEAAATAAAAGLMMMRRMRRAPAEAAEAPPQSE
P10_UL112 PSSTSPSSLLSLPRPSSAHSAGETVQESEAAATAAAAGLMMMRRMRRAPAEAAEAPPQSE

P4_UL112 EENDSTTPVSNCRVPPNSQESAAPQPPRSPRFDDIIQSLTKMLNDCKEKRLCDLPLVSSR
P6_UL112 EENDSTTPVSNCRVPPNSQESAAPQPPRSPRFDDIIQSLTKMLNDCKEKRLCDLPLVSSR
P14_UL112 EENDSTTPVSNCRVPPNSQESAAPQPPRSPRFDDIIQSLTKMLNDCKEKRLCDLPLVSSR
P15_UL112 EENDSTTPVSNCRVPPNSQESAAPQPPRSPRFDDIIQSLTKMLNDCKEKRLCDLPLVSSR
P10_UL112 EENDSTTPVSNCRVPPNSQESAAPQPPRSPRFDDIIQSLTKMLNDCKEKRLCDLPLVSSR

P4_UL112 LLPETSGGTVVVNHSSVARTAAAVSTAGVGPPAAACPPLVTTGVVPSGSVAGVAPVAAAV
P6_UL112 LLPETSGGTVVVNHSSVARTAAAVSTAGVGPPAAACPPLVTTGVVPSGSVAGVAPVAAAV
P14_UL112 LLPETSGGTVVVNHSSVARTAAAVSTAGVGPPAAACPPLVTTGVVPSGSVAGVAPVAAAV
P15_UL112 LLPETSGGTVVVNHSSVARTAAAVSTAGVGPPAAACPPLVTTGVVPSGSVAGVAPVAAAV
P10_UL112 LLPETSGGTVVVNHSSVARTAAAVSTAGVGPPAAACPPLVTTGVVPSGSVAGVAPVAAAV

P4_UL112 ETPAAPPRPVCEIKPYVVNPVVATAAAASNSSSSSSTPLPPPPPPPGGRRGRARNNTRGG
P6_UL112 ETPAAPPRPVCEIKPYVVNPVVATAAAASNSSSSSSTPLPPPPPPPGGRRGRARNNTRGG
P14_UL112 ETPAAPPRPVCEIKPYVVNPVVATAAAASNSSSSSSTPLPPPPPPPGGRRGRARNNTRGG
P15_UL112 ETPAAPPRPVCEIKPYVVNPVVATAAAASNSSSSSSTPLPPPPPPPGGRRGRARNNTRGG
P10_UL112 ETPAAPPRPVCEIKPYVVNPVVATAAAASNSSSSSSAPLPPPPPPPGGRRGRARNNTRGG

P4_UL112 GGGGGGRNSRRQAASSSSSSSRRSRRRNNRHEDEDNDPLLRLSQVAGSGRRRGPSFLEDG
P6_UL112 GGGGGGRNSRRQAASSSSSSSRRSRRRNNRHEDEDNDPLLRLSQVAGSGRRRGPSFLEDG
P14_UL112 GGGGGGRNSRRQAASSSSSSSRRSRRRNNRHEDEDNDPLLRLSQVAGSGRRRGPSFLEDG
P15_UL112 GGGGGGRNSRRQAASSSSSSSRRSRRRNNRHEDEDNDPLLRLSQVAGSGRRRGPSFLEDG
P10_UL112 GGGGGGRNSRRQAASSSSSSSRRSRRRNNRHEDEDNDPLLRLSQVAGSGRRRGPSFLEDG

P4_UL112 LEIIDPSEEAAIAAASIAAFFDD
P6_UL112 LEIIDPSEEAAIAAASIAAFFDD
P14_UL112 LEIIDPSEEAAIAAASIAAFFDD
P15_UL112 LEIIDPSEEAAIAAASIAAFFDD
P10_UL112 LEIIDPSEEAAIAAASIAAFFDD

UL119

P4_UL119 MCPVLAIVLVVALLGDTHPGVESSTTSAVTSPSNTTATSTTSISTSNNVTSAVTTTVQTS
P6_UL119 MCPVLAIVLVVALLGDTHPGVESSTTSAVTSPSNTTATSTTSISTSNNVTSAVTTTVQTS
P14_UL119 MCPVLAIVLVVALLGDTHPGVESSTTSAVTSPSNTTATSTTSISTSNNVTSAVTTTVQTS
P15_UL119 MCPVLAIVLVVALLGDTHPGVESSTTSAVTSPSNTTATSTTSISTSNNVTSAVTTTVQTS
P10_UL119 MCSVLAIALVVALLGDMHPGVKSSTTSAVTSPSNTTVTSTTSISTSNNVSSAVTTTVQTS

P4_UL119 TSSASTSVIATTQKEGRLYTVNCEASYSYDQVSLNATCKVILLNNTKNPDILSVTCYART
P6_UL119 TSSASTSVIATTQKEGRLYTVNCEASYSYDQVSLNATCKVILLNNTKNPDILSVTCYART
P14_UL119 TSSASTSVIATTQKEGRLYTVNCEASYSYDQVSLNATCKVILLNNTKNPDILSVTCYART
P15_UL119 TSSASTSVIATTQKEGRLYTVNCEASYSYDQVSLNATCKVILLNNTKNPDILSVTCYART
P10_UL119 TSSASTSVIATTQKEGHLYTVNCEASYSYDQVSLNATCKVILLNNTKNPDILSVTCYART

P4_UL119 DCKGPFTQVGYLSAFPPDNEGKLHLSYNATAQELLISGLRPQETTEYTCSFFSWGRHHNA
P6_UL119 DCKGPFTQVGYLSAFPPDNEGKLHLSYNATAQELLISGLRPQETTEYTCSFFSWGRHHNA
P14_UL119 DCKGPFTQVGYLSAFPPDNEGKLHLSYNATAQELLISGLRPQETTEYTCSFFSWGRHHNA
P15_UL119 DCKGPFTQVGYLSAFPPDNEGKLHLSYNATAQELLISGLRPQETTEYTCSFFSWGRHHNA
P10_UL119 DCKGPFTQVGYLSAFPSNDKGKLHLSYNATAQELLISGLRPQETTEYTCSFFSWGRHHNA

P4_UL119 TWDLFTYPIYAVYGTRLNATTMRVRVLLQEHEHCLLNGSSLYHPNSTVHLHQGNQLIPPW
P6_UL119 TWDLFTYPIYAVYGTRLNATTMRVRVLLQEHEHCLLNGSSLYHPNSTVHLHQGNQLIPPW
P14_UL119 TWDLFTYPIYAVYGTRLNATTMRVRVLLQEHEHCLLNGSSLYHPNSTVHLHQGNQLIPPW
P15_UL119 TWDLFTYPIYAVYGTRLNATTMRVRVLLQEHEHCLLNGSSLYHPNSTVHLHQGNQLIPPW
P10_UL119 TWDLFTYPIYAVYGTRLNATTMRVRVLLQEHEHCLLNGSSLYHPNSTVHLHQGDQLIPPW

P4_UL119 NISNVTYNGQRLREFVFYLNGTYTVVRLHVQIAGRSFTTTYVFIKSDPLFEDRLLAYGVL
P6_UL119 NISNVTYNGQRLREFVFYLNGTYTVVRLHVQIAGRSFTTTYVFIKSDPLFEDRLLAYGVL
P14_UL119 NISNVTYNGQRLREFVFYLNGTYTVVRLHVQIAGRSFTTTYVFIKSDPLFEDRLLAYGVL
P15_UL119 NISNVTYNGQRLREFVFYLNGTYTVVRLHVQIAGRSFTTTYVFIKSDPLFEDRLLAYGVL
P10_UL119 NISNVTYNGQRLREFVFYLNGTYTVVRLHVQIAGRSFTTTYVFIKSDPLFEDRLLAYGVL

P4_UL119 AFLVFMVIILLYVTYMLARRRDWSYKRLEEPVEEKKHPVPYFKQW
P6_UL119 AFLVFMVIILLYVTYMLARRRDWSYKRLEEPVEEKKHPVPYFKQW
P14_UL119 AFLVFMVIILLYVTYMLARRRDWSYKRLEEPVEEKKHPVPYFKQW
P15_UL119 AFLVFMVIILLYVTYMLARRRDWSYKRLEEPVEEKKHPVPYFKQW
P10_UL119 AFLVFMVIILLYVTYMLARRRDWSYKRLEEPVEEKKHPVPYFKQW

UL135

P4_uL135 MVWLWLGVGLLGGTGLASLVLAISLFTQRRGRKRSDETSSRGRLPGAASDKRGACACCYR
P6_UL135 MVWLWLGVGLLGGTGLASLVLAISLFTQRRGRKRSDETSSRGRLPGAASDKRGACACCYR
P10_UL135 MVWLWLGVGLLGGTGLASLVLAISLFTQRRGRKRSDETSSRGRLPGAASDKRGACACCYR
P14_UL135 MVWLWLGVGLLGGTGLASLVLAISLFTQRRGRKRSDETSSRGRLPGAASDKRGACACCYR
P15_UL135 MVWLWLGVGLLGGTGLASLVLAISLFTQRRGRKRSDETSSRGRLPGAASDKRGACACCYR

P4_uL135 IPKEDVVEPLDLELGLMRVATHPPTPQVPRCTSLYIGEDGLPIDKPEFPPARFEIPDVST
P6_UL135 IPKEDVVEPLDLELGLMRVATHPPTPQVPRCTSLYIGEDGLPIDKPEFPPARFEIPDVST
P10_UL135 IPKEDVVEPLDLELGLMRVATHPPTPQVPRCTSLYIGEDGLPIDKPEFPPARFEIPDVST
P14_UL135 IPKEDVVEPLDLELGLMRVATHPPTPQVPRCTSLYIGEDGLPIDKPEFPPARFEIPDVST
P15_UL135 IPKEDVVEPLDLELGLMRVATHPPTPQVPRCTSLYIGEDGLPIDKPEFPPARFEIPDVST

P4_uL135 PGTPTSIGRSPSHCSSSSSLSSSASVDTVLHQPPPSWKPPPPPGRKKRPPTPPVRAPTTR
P6_UL135 PGTPTSIGRSPSHCSSSSSLSSSASVDTVLHQPPPSWKPPPPPGRKKRPPTPPVRAPTTR
P10_UL135 PGTPTSIGRSPSHCSSSSSLSSSASVDTVLHQPPPSWKPPPPPGRKKRPPTPPVRAPTTR
P14_UL135 PGTPTSIGRSPSHCSSSSSLSSSASVDTVLHQPPPSWKPPPPPGRKKRPPTPPVRAPTTR
P15_UL135 PGTPTSIGRSPSHCSSSSSLSSSASVDTVLHQPPPSWKPPPPPGRKKRPPTPPVRAPTTR

P4_uL135 LSSHRPPTPIPAPRKNLSTPPTKKTPPPTKPKPVGWTPPVTPRPFPKTPTPQKPPRNPRL
P6_UL135 LSSHRPPTPIPAPRKNLSTPPTKKTPPPTKPKPVGWTPPVTPRPFPKTPTPQKPPRNPRL
P10_UL135 LSSHRPPTPIPAPRKNLSTPPTKKTPPPTKPKPVGWTPPVTPRPFPKTPTPQKPPRNPRL
P14_UL135 LSSHRPPTPIPAPRKNLSTPPTKKTPPPTKPKPVGWTPPVTPRPFPKTPTPQKPPRNPRL
P15_UL135 LSSHRPPTPIPAPRKNLSTPPTKKTPPPTKPKPVGWTPPVTPRPFPKTPTPQKPPRNPRL

P4_uL135 PRTVGLENLSKVGLSCPCPRPRTPTEPTTLPIVSVSELAPPPRWSDIEELLEKAVQSVMK
P6_UL135 PRTVGLENLSKVGLSCPCPRPRTPTEPTTLPIVSVSELAPPPRWSDIEELLEKAVQSVMK
P10_UL135 PRTVGLENLSKVGLSCPCPRPRTPTEPTTLPIVSVSELAPPPRWSDIEELLEKAVQSVMK
P14_UL135 PRTVGLENLSKVGLSCPCPRPRTPTEPTTLPIVSVSELAPPPRWSDIEELLEKAVQSVMK
P15_UL135 PRTVGLENLSKVGLSCPCPRPRTPTEPTTLPIVSVSELAPPPRWSDIEELLEKAVQSVMK

P4_uL135 DAESMQMT
P6_UL135 DAESMQMT
P10_UL135 DAESMQMT
P14_UL135 DAESMQMT
P15_UL135 DAESMQMT

UL141

P6_UL141 MCRRESLRTLPWLFWVLLSCPRLLEYSSSSFPFATADIAEKMWAENYETTSPAPVLVAEG
P10_UL141 MCRRESLRTLPWLFWVLLSCPRLLEYSSSSFPFATADIAEKMWAENYETTSPAPVLVAEG

P6_UL141 EQVTIPCTVMTHSWPMVSIRARFCRSHDGSDELILDAVKGHRLMNGLQYRLPYATWNFSQ
P10_UL141 EQVTIPCTVMTHSWPMVSIRARFCRSHDGSDELILDAVKGHRLMNGLQYRLPYATWNFSQ

P6_UL141 LHLGQIFSLTFNVSTDTAGMYECVLRNYSHGLIMQRFVILTQLETLSRPDEPCCTPALGR
P10_UL141 LHLGQIFSLTFNVSTDTAGMYECVLRNYSHGLIMQRFVILTQLETLSRPDEPCCTPALGR

P6_UL141 YSLGDQIWSPTPWRLRNHDCGMYRGFQRNYFYIGRADAEDCWKPACPDEEPDRCWTVIQR
P10_UL141 YSLGDQIWSPTPWRLRNHDCGMYRGFQRNYFYIGRADAEDCWKPACPDEEPDRCWTVIQR

```
P6_UL141        YRLPGDCYRSQPHPPKFLPVTPAPPADIDTGMSPWATRGIAAFLGFWSIFTVCFLCYLCY
P10_UL141       YRLPGDCYRSQPHPPKFLPVTPAPPADIDTGMSPWATRGIAAFLGFWSIFTVCFLCYLCY

P6_UL141        LQCCGRWCPTPGRGRRGGEGYRRLPTYDSYPGVKKMKR
P10_UL141       LQCCGRWCPTPGRGRRGGEGYRRLPTYDSYPGVKKMKR
```

UL142

```
P6_UL142        MRIEWACWLFGYFVSSVGSERSLSYRYHLESNSSANVVCNGNISVFVNGTLGVRYNITVG
P10_UL142       MRIEWACWLFGYFVSSVGSERSLSYRYHLESNSSANVVCNGNISVFVNGTLGVRYNITVG


P6_UL142        ISSSLLIGHLTIQTLESWFTSWVQNKSYSKQPLSTTETLYNIDSENIHRVSQYFHTRWIK
P10_UL142       ISSSLLIGHLTIQTLESWFTSWVQNKSYSKQPLSTTETLYNIDSENIHRVSQYFHTRWIK

P6_UL142        SLQENHTCDLTNSTPTYTYQANVNNTNYLTLTSSGWQDRLNYTAINSTHFNLTESNITSI
P10_UL142       SLQENHTCDLTNSTPTYTYQANVNNTNYLTLTSSGWQDRLNYTAINSTHFNLTESNITSI

P6_UL142        HKYLNTTCIERLRNYTLKSVYTTTVPQNVTTPQHTTTTLYTPPNAITIQDTTQSHTVQTP
P10_UL142       HKYLNTTCIERLRNYTLKSVYTTTVPQNVTTPQHTTTTLYTPPNAITIQDTTQSHTVQTP

P6_UL142        SFNDTHNVTEHTLNINYVLSQKTNNTTSPWVYAIPMGATATIGASLYIGKHFTPVRFVYE
P10_UL142       SFNDTHNVTEHTLNINYVLSQKTNNTTSPWVYAIPMGATATIGASLYIGKHFTPVRFVYE

P6_UL142        VWRGQ
P10_UL142       VWRGQ
```

UL148

```
P4_UL148        MLRLLFTLVLLALHGPSVNASRDYVHVRLLSYRGDPLVFKHTFSGVRRPFTELGWAVCRD
P6_UL148        MLRLLFTLVLLALHGPSVNASRDYVHVRLLSYRGDPLVFKHTFSGVRRPFTELGWAVCRD
P14_UL148       MLRLLFTLVLLALHGPSVNASRDYVHVRLLSYRGDPLVFKHTFSGVRRPFTELGWAVCRD
P15_UL148       MLRLLFTLVLLALHGPSVNASRDYVHVRLLSYRGDPLVFKHTFSGVRRPFTELGWAVCRD
P10_UL148       MLRLLFTLVLLALHGPSVNASRDYVHVRLLSYRGDPLVFKHTFSGVRRPFTELGWAACRD

P4_UL148        WDSMHCTPFWSTDLEQMTDSVRRYSTVSPGKEVTLQLHGNQTVQPSFLSFTCRLQLEPVV
P6_UL148        WDSMHCTPFWSTDLEQMTDSVRRYSTVSPGKEVTLQLHGNQTVQPSFLSFTCRLQLEPVV
P14_UL148       WDSMHCTPFWSTDLEQMTDSVRRYSTVSPGKEVTLQLHGNQTVQPSFLSFTCRLQLEPVV
P15_UL148       WDSMHCTPFWSTDLEQMTDSVRRYSTVSPGKEVTLQLHGNQTVQPSFLSFTCRLQLEPVV
P10_UL148       WDSMHCTPFWSTDPEQMTDSVRRYSTVSPGKEVTLQLHGNQTVQPSFLSFTCRLQLEPVV

P4_UL148        ENVGLYVAYVVNDGERPQQFFTPQVDVVRFALYLETLSRIVEPLESGRLAVEFDTPDLAL
P6_UL148        ENVGLYVAYVVNDGERPQQFFTPQVDVVRFALYLETLSRIVEPLESGRLAVEFDTPDLAL
P14_UL148       ENVGLYVAYVVNDGERPQQFFTPQVDVVRFALYLETLSRIVEPLESGRLAVEFDTPDLAL
P15_UL148       ENVGLYVAYVVNDGERPQQFFTPQVDVVRFALYLETLSRIVEPLESGRLAVEFDTPDLAL
P10_UL148       ENVGLYVAYVVNDGERPQQFFTPQVDVVRFALYLETLSRIVEPLESGRLAVEFDTPDLAL

P4_UL148        APDLVSSLFVAGHGETDFYMNWTLRRSQTHYLEEMALQVEILKPRGVRHRAIIHHPKLQP
P6_UL148        APDLVSSLFVAGHGETDFYMNWTLRRSQTHYLEEMALQVEILKPRGVRHRAIIHHPKLQP
P14_UL148       APDLVSSLFVAGHGETDFYMNWTLRRSQTHYLEEMALQVEILKPRGVRHRAIIHHPKLQP
P15_UL148       APDLVSSLFVAGHGETDFYMNWTLRRSQTHYLEEMALQVEILKPRGVRHRAIIHHPKLQP
P10_UL148       APDLVSSLFVAGHGETDFYMNWTLRRSQTHYLEEMALQVEILKPRGVRHRAIIHHPKLQP

P4_UL148        GVGLWIDFCVYRYNARLTRGYVRYTLSPKARLPAKAEGWLVSLDRFIVQYLNTLLITMMA
P6_UL148        GVGLWIDFCVYRYNARLTRGYVRYTLSPKARLPAKAEGWLVSLDRFIVQYLNTLLITMMA
P14_UL148       GVGLWIDFCVYRYNARLTRGYVRYTLSPKARLPAKAEGWLVSLDRFIVQYLNTLLITMMA
P15_UL148       GVGLWIDFCVYRYNARLTRGYVRYTLSPKARLPAKAEGWLVSLDRFIVQYLNTLLITMMA
P10_UL148       GVGLWIDFCVYRYNARLTRGYVRYTLSPKARLPAKAEGWLVSLDRFIVQYLNTLLITMMA

P4_UL148        AIWARVLITYLVSRRR
P6_UL148        AIWARVLITYLVSRRR
P14_UL148       AIWARVLITYLVSRRR
P15_UL148       AIWARVLITYLVSRRR
P10_UL148       AIWARVLITYLVSRRR
```

UL148A

```
P4_UL148A       MSSDSNFDPWIPVCVVVVMTSVVLFAGLHVYLWYVRRQLVAFCLEKVCVRCCGKDETTPL
P6_UL148A       MSSDSNFDPWIPVCVVVVMTSVVLFAGLHVYLWYVRRQLVAFCLEKVCVRCCGKDETTPL
P14_UL148A      MSSDSNFDPWIPVCVVVVMTSVVLFAGLHVYLWYVRRQLVAFCLEKVCVRCCGKDETTPL
P15_UL148A      MSSDSNFDPWIPVCVVVVMTSVVLFAGLHVYLWYVRRQLVAFCLEKVCVRCCGKDETTPL
P10_UL148A      MSSSDNLDPWIPVCVVVVMTSVVLFAGLHVYLWYVRRQLVAFCLEKVCVRCCGKDETTPL

P4_UL148a       VEDAEPPAELEMVEVSDECY
P6_UL148A       VEDAEPPAELEMVEVSDECY
P14_UL148A      VEDAEPPAELEMVEVSDECY
P15_UL148A      VEDAEPPAELEMVEVSDECY
P10_UL148A      VEDAEPPAELEMVEVSDECY
```

US9

```
P4_US9          MILWSPSTCSFFWHWCLIAVSVLSSRSKESLRLSWSSDESSASSSSRICPLSNSKSVRLP
P6_US9          MILWSPSTCSFFWHWCLIAVSVLSSRSKESLRLSWSSDESSASSSSRICPLSNSKSVRLP
P10_US9         MILWSPSTCSFFWHWCLIAVSVLSSRSKESLRLSWSSDESSASSSSRICPLSNSKSVRLP
P14_US9         MILWSPSTCSFFWHWCLIAVSVLSSRSKESLRLSWSSDESSASSSSRICPLSNSKSVRLP
P15_US9         MILWSPSTCSFFWHWCLIAVSVLSSRSKESLRLSWSSDESSASSSSRICPLSNSKSVRLP

P4_US9          QYPRGFGDVSGYRVSSSVSECYVQHGVLVAAWLVRGNFSDTAPRAYGTWGNERSATHFKV
P6_US9          QYPRGFGDVSGYRVSSSVSECYVQHGVLVAAWLVRGNFSDTAPRAYGTWGNERSATHFKV
P10_US9         QYPRGFGDVSGYRVSSSVSECYVQHGVLVAAWLVRGNFSDTAPRAYGTWGNERSATHFKV
P14_US9         QYPRGFGDVSGYRVSSSVSECYVQHGVLVAAWLVRGNFSDTAPRAYGTWGNERSATHFKV
P15_US9         QYPRGFGDVSGYRVSSSVSECYVQHGVLVAAWLVRGNFSDTAPRAYGTWGNERSATHFKV

P4_US9          GAPQLENDGALRYETELPQVDARLSYVMLTVYPCSACNRSVLHCRPASRLPWLPLRVTPS
P6_US9          GAPQLENDGALRYETELPQVDARLSYVMLTVYPCSACNRSVLHCRPASRLPWLPLRVTPS
P10_US9         GAPQLENDGALRYETELPQVDARLSYVMLTVYPCSACNRSVLHCRPASRLPWLPLRVTPS
P14_US9         GAPQLENDGALRYETELPQVDARLSYVMLTVYPCSACNRSVLHCRPASRLPWLPLRVTPS
P15_US9         GAPQLENDGALRYETELPQVDARLSYVMLTVYPCSACNRSVLHCRPASRLPWLPLRVTPS

P4_US9          DLERLFAERRYLTFLYVVLVQFVKHVALFSFGVQVACCVYLRWIRPWVRGRHRATGRTSR
P6_US9          DLERLFAERRYLTFLYVVLVQFVKHVALFSFGVQVACCVYLRWIRPWVRGRHRATGRTSR
P10_US9         DLERLFAERRYLTFLYVVLVQFVKHVALFSFGVQVACCVYLRWIRPWVRGRHRATGRTSR
P14_US9         DLERLFAERRYLTFLYVVLVQFVKHVALFSFGVQVACCVYLRWIRPWVRGRHRATGRTSR
P15_US9         DLERLFAERRYLTFLYVVLVQFVKHVALFSFGVQVACCVYLRWIRPWVRGRHRATGRTSR

P4_US9          EEEAKDD
P6_US9          EEEAKDD
P10_US9         EEEAKDD
P14_US9         EEEAKDD
P15_US9         EEEAKDD
```

US12

```
P4_US12         MVQIQFHQGEPLGHKKEKPPPVSPPSPPPIRRVTVITKDEDTLRSVQHFLWMVRLYGTVV
P6_US12         MVQIQFHQGEPLGHKKEKPPPVSPPSPPPIRRVTVITKDEDTLRSVQHFLWMVRLYGTVV
P10_US12        MVQIQFHQGEPLGHKKEKPPPVSPPSPPPIRRVTVITKDEDTLRSVQHFLWMVRLYGTVV
P15_US12        MVQIQFHQGEPLGHKKEKPPPVSPPSPPPIRRVTVITKDEDTLRSVQHFLWMVRLYGTVV
P14_US12        MVQIQFHQGEPLGHKKEKPPPVSPPSPPPIRRVTVITKDEDTLRSVQHFLWMVRLYGTVV

P4_US12         FQTSATIATTILFMLIPWRVTTPYLRDTLPFWSTLLPCALRCHAYWLERRRRPGTLMLVM
P6_US12         FQTSATIATTILFMLIPWRVTTPYLRDTLPFWSTLLPCALRCHAYWLERRRRPGTLMLVM
P10_US12        FQTSATIATTILFMLIPWRVTTPYLRDTLPFWSTLLPCALRCHAYWLERRRRPGTLMLVM
P15_US12        FQTSATIATTILFMLIPWRVTTPYLRDTLPFWSTLLPCALRCHAYWLERRRRPGTLMLVM
P14_US12        FQTSATIATTILFMLIPWRVTTPYLRDTLPFWSTLLPCALRCHAYWLERRRRPGTLMLVM

P4_US12         VYTTLTTISVSTIGLCFDRTVVIQAYVLSSMLCVWCTGLAWLMAWNMQRRLAILCLLSFM
P6_US12         VYTTLTTISVSTIGLCFDRTVVIQAYVLSSMLCVWCTGLAWLMAWNMQRRLAILCLLSFM
P10_US12        VYTTLTTISVSTIGLCFDRTVVIQAYVLSSMLCVWCTGLAWLMAWNMQRRLAILCLLSFM
P15_US12        VYTTLTTISVSTIGLCFDRTVVIQAYVLSSMLCVWCTGLAWLMAWNMQRRLAILCLLSFM
P14_US12        VYTTLTTISVSTIGLCFDRTVVIQAYVLSSMLCVWCTGLAWLMAWNMQRRLAILCLLSFM

P4_US12         LPILWLFIAVQSWEPYQRIILALTVSFIYGLKIVLIRDTLTVLYRSPSNCYTDGDLLRTA
P6_US12         LPILWLFIAVQSWEPYQRIILALTVSFIYGLKIVLIRDTLTVLYRSPSNCYTDGDLLRTA
P10_US12        LPILWLFIAVQSWEPYQRIILALTVSFIYGLKIVLIRDTLTVLYRSPSNCYTDGDLLRTA
P15_US12        LPILWLFIAVQSWEPYQRIILALTVSFIYGLKIVLIRDTLTVLYRSPSNCYTDGDLLRTA
```

```
P14_US12        LPILWLFIAVQSWEPYQRIILALTVSFIYGLKIVLIRDTLTVLYRSPSNCYTDGDLLRTA

P4_US12         MLLYMDQVIMFLLVVVPLTAPIWYPNYAGALGRTAHWLFHK
P6_US12         MLLYMDQVIMFLLVVVPLTAPIWYPNYAGALGRTAHWLFHK
P10_US12        MLLYMDQVIMFLLVVVPLTAPIWYPNYAGALGRTAHWLFHK
P15_US12        MLLYMDQVIMFLLVVVPLTAPIWYPNYAGALGRTAHWLFHK
P14_US12        MLLYMDQVIMFLLVVVPLTAPIWYPNYAGALGRTAHWLFHK
```

US14

```
P14_US14        METVSTQRETASSETERTREAASAETTDATFRSLEEGSTISSRYSETASTVSEDAVCWLR
P15_US14        METVSTQRETASSETERTREAASAETTDATFRSLEEGSTISSRYSETASTVSEDAVCWLR
P10_US14        MKTVSTQRETASSETERTREAASAETTDATFRSLEEGSTISSRYSETASTVSENAVCWLR
P6_US14         METVSTQRETASSETERTREAASAETTDATFRSLEEGSTISSRYSETASTVSEDAVCWLR
P4_US14         METVSTQRETASSETERTREAASAETTDATFRSLEEGSTISSRYSETASTVSEDAVCWLR

P14_US14        RTAIVMRVYGLLTLETAFSVLISALVWLGYPSLGYECSDDPSPLLLSCTPVLVLGALELT
P15_US14        RTAIVMRVYGLLTLETAFSVLISALVWLGYPSLGYECSDDPSPLLLSCTPVLVLGALELT
P10_US14        RTAIVMRVYGLLTLETAFSVLISALVWLGYPSLGYECSDDPSPLLLSCTPVLVLGALELT
P6_US14         RTAIVMRVYGLLTLETAFSVLISALVWLGYPSLGYECSDDPSPLLLSCTPVLVLGALELT
P4_US14         RTAIVMRVYGLLTLETAFSVLISALVWLGYPSLGYECSDDPSPLLLSCTPVLVLGALELT

P14_US14        DHRHPSNGLVFALYVALLSFTTAGLNLCATAPIGISSLILTWTLFVACNGVAWEHRLSSV
P15_US14        DHRHPSNGLVFALYVALLSFTTAGLNLCATAPIGISSLILTWTLFVACNGVAWEHRLSSV
P10_US14        DHRHPSNGLVFALYVALLSFTTAGLNLCATAPIGISSLILTWTLFVACNGVAWEHRLSSV
P6_US14         DHRHPSNGLVFALYVALLSFTTAGLNLCATAPIGISSLILTWTLFVACNGVAWEHRLSSV
P4_US14         DHRHPSNGLVFALYVALLSFTTAGLNLCATAPIGISSLILTWTLFVACNGVAWEHRLSSV

P14_US14        WRDALFTSTLLTVMVSVLASTYTWLHKTLLCLYTVFVGCILAVLFQDVRYIATKMPVSHV
P15_US14        WRDALFTSTLLTVMVSVLASTYTWLHKTLLCLYTVFVGCILAVLFQDVRYIATKMPVSHV
P10_US14        WRDALFTSTLLTVMVSVLASTYTWLHKTLLCLYTVFVGCILAVLFQDVRYIATKMPVNHV
P6_US14         WRDALFTSTLLTVMVSVLASTYTWLHKTLLCLYTVFVGCILAVLFQDVRYIATKMPVSHV
P4_US14         WRDALFTSTLLTVMVSVLASTYTWLHKTLLCLYTVFVGCILAVLFQDVRYIATKMPVSHV

P14_US14        IRSSLILYATETLIYHTTLLMLTPVVWSARWDQMFSYLAKLGTYHHYLIDNGTLSVILNT
P15_US14        IRSSLILYATETLIYHTTLLMLTPVVWSARWDQMFSYLAKLGTYHHYLIDNGTLSVILNT
P10_US14        IRSSLILYATETLIYHTTLLMLTPVVWSARWDQMFSYLAKLGTYHHYLIDNGTLSVILNS
P6_US14         IRSSLILYATETLIYHTTLLMLTPVVWSARWDQMFSYLAKLGTYHHYLIDNGTLSVILNT
P4_US14         IRSSLILYATETLIYHTTLLMLTPVVWSARWDQMFSYLAKLGTYHHYLIDNGTLSVILNT

P14_US14        TTATFQSKAA
P15_US14        TTATFQSKAA
P10_US14        TTATFQSRVA
P6_US14         TTATFQSKAA
P4_US14         TTATFQSKAA
```

US18

```
P4_US18         MGDTASVSEHHESPTVTIVPLHRSHALVAEQQLFQWLKRFKLLMEVYHGLVWQLACTLTV
P6_US18         MGDTASVSEHHESPTVTIVPLHRSHALVAEQQLFQWLKRFKLLMEVYHGLVWQLACTLTV
P10_US18        MGDTASVSEHHESPTVTIVPLHRSHALVAEQQLFQWLKRFKLLMEVYHGLVWQLACTLTV
P14_US18        MGDTASVSEHHESPTVTIVPLHRSHALVAEQQLFQWLKRFKLLMEVYHGLVWQLACTLTV
P15_US18        MGDTASVSEHHESPTVTIVPLHRSHALVAEQQLFQWLKRFKLLMEVYHGLVWQLACTLTV

P4_US18         CLLAWLAFPDVQGQCANGIVPALSSIVPVSTLAMLRGFAEFRPHTTNFAHLTVACLLINT
P6_US18         CLLAWLAFPDVQGQCANGIVPALSSIVPVSTLAMLRGFAEFRPHTTNFAHLTVACLLINT
P10_US18        CLLAWLAFPDVQGQCANGIVPALSSIVPVSTLAMLRGFAEFRPHTTNFAHLTVACLLINT
P14_US18        CLLAWLAFPDVQGQCANGIVPALSSIVPVSTLAMLRGFAEFRPHTTNFAHLTVACLLINT
P15_US18        CLLAWLAFPDVQGQCANGIVPALSSIVPVSTLAMLRGFAEFRPHTTNFAHLTVACLLINT

P4_US18         GITVCTGFCGERRVIGLSFALVMVFFVLCSGLTYLAGNNPTRWKVIGIGYGWSVIVFYLL
P6_US18         GITVCTGFCGERRVIGLSFALVMVFFVLCSGLTYLAGNNPTRWKVIGIGYGWSVIVFYLL
P10_US18        GITVCTGFCGERRVIGLSFALVMVFFVLCSGLTYLAGNNPTRWKVIGIGYGWSVIVFYLL
P14_US18        GITVCTGFCGERRVIGLSFALVMVFFVLCSGLTYLAGNNPTRWKVIGIGYGWSVIVFYLL
P15_US18        GITVCTGFCGERRVIGLSFALVMVFFVLCSGLTYLAGNNPTRWKVIGIGYGWSVIVFYLL

P4_US18         LYFSPVLWVSKIYSGLYVLVVTAASAVLIYETLDLIYQRGTLSKNSVCVSVVLYTIVMSL
P6_US18         LYFSPVLWVSKIYSGLYVLVVTAASAVLIYETLDLIYQRGTLSKNSVCVSVVLYTIVMSL
```

```
P10_US18        LYFSPVLWVSKIYSGLYVLVVTAASAVLIYETLDLIYQRGTLSKNSVCVSVVLYTIVMSL
P14_US18        LYFSPVLWVSKIYSGLYVLVVTAASAVLIYETLDLIYQRGTLSKNSVCVSVVLYTIVMSL
P15_US18        LYFSPVLWVSKIYSGLYVLVVTAASAVLIYETLDLIYQRGTLSKNSVCVSVVLYTIVMSL

P4_US18         LNMSVAIFSGHVWVQQYAEKHGGRIDGVSLLSLL
P6_US18         LNMSVAIFSGHVWVQQYAEKHGGRIDGVSLLSLL
P10_US18        LNMSVAIFSGHVWVQQYAEKHGGRIDGVSLLSLL
P14_US18        LNMSVAIFSGHVWVQQYAEKHGGRIDGVSLLSLL
P15_US18        LNMSVAIFSGHVWVQQYAEKHGGRIDGVSLLSLL
```

US20

```
P4_US20         MQAQEANALLLSRMEALEWFKKFTVWLRVYAIFIFQLAFSFGLGSVFWLGFPQNRNFCVE
P6_US20         MQAQEANALLLSRMEALEWFKKFTVWLRVYAIFIFQLAFSFGLGSVFWLGFPQNRNFCVE
P10_US20        MQAQEANALLLSRMEALEWFKKFTVWLRVYAIFIFQLAFSFGLGSVFWLGFPQNRNFCVE
P14_US20        MQAQEANALLLSRMEALEWFKKFTVWLRVYAIFIFQLAFSFGLGSVFWLGFPQNRNFCVE
P15_US20        MQAQEANALLLSRMEALEWFKKFTVWLRVYAIFIFQLAFSFGLGSVFWLGFPQNRNFCVE

P4_US20         NYSFFLTVLVPIVCMFITYTLGNEHPSNATVLFIYLLANSLTAAIFQMCSESRVLVGSYV
P6_US20         NYSFFLTVLVPIVCMFITYTLGNEHPSNATVLFIYLLANSLTAAIFQMCSESRVLVGSYV
P10_US20        NYSFFLTVLVPIVCMFITYTLGNEHPSNATVLFIYLLANSLTAAIFQMCSESRVLVGSYV
P14_US20        NYSFFLTVLVPIVCMFITYTLGNEHPSNATVLFIYLLANSLTAAIFQMCSESRVLVGSYV
P15_US20        NYSFFLTVLVPIVCMFITYTLGNEHPSNATVLFIYLLANSLTAAIFQMCSESRVLVGSYV

P4_US20         MTLALFISFTGLAFLGGRDRRRWKCISCVYVVMLLSFLTLALLSDADWLQKIVVTLCAFS
P6_US20         MTLALFISFTGLAFLGGRDRRRWKCISCVYVVMLLSFLTLALLSDADWLQKIVVTLCAFS
P10_US20        MTLALFISFTGLAFLGGRDRRRWKCISCVYVVMLLSFLTLALLSDADWLQKIVVTLCAFS
P14_US20        MTLALFISFTGLAFLGGRDRRRWKCISCVYVVMLLSFLTLALLSDADWLQKIVVTLCAFS
P15_US20        MTLALFISFTGLAFLGGRDRRRWKCISCVYVVMLLSFLTLALLSDADWLQKIVVTLCAFS

P4_US20         ISFFLGILAYDSLMVIFFCPPNQCIRHAVCLYLDSMAIFLTLLLMLSGPRWISLSDGAPL
P6_US20         ISFFLGILAYDSLMVIFFCPPNQCIRHAVCLYLDSMAIFLTLLLMLSGPRWISLSDGAPL
P10_US20        ISFFLGILAYDSLMVIFFCPPNQCIRHAVCLYLDSMAIFLTLLLMLSGPRWISLSDGAPL
P14_US20        ISFFLGILAYDSLMVIFFCPPNQCIRHAVCLYLDSMAIFLTLLLMLSGPRWISLSDGAPL
P15_US20        ISFFLGILAYDSLMVIFFCPPNQCIRHAVCLYLDSMAIFLTLLLMLSGPRWISLSDGAPL

P4_US20         DNGTLTAASTTGKS
P6_US20         DNGTLTAASTTGKS
P10_US20        DNGTLTAASTTGKS
P14_US20        DNGTLTAASTTGKS
P15_US20        DNGTLTAASTTGKS
```
