# Supplementary material for: Genetic Variability of Human Cytomegalovirus Clinical Isolates Correlates With Altered Expression of Natural Killer Cell-Activating Ligands and IFN-γ
Source: Front Immunol. 2021 Apr 9;12:532484. doi: 10.3389/fimmu.2021.532484 (PMC8062705; doi:10.3389/fimmu.2021.532484)
Supplement: Supplementary file 5 [file Table_1.pdf]

Supplementary Table 1: Summary of sequencing data

**Patient**	**P4**	**P6**	**P10**	**P14**	**P15**
Total trimmed reads (no.)	462,212	7,083,940	1,207,286	540,480	386,112
Duplicates (%)	0.17	0.25	0.41	0.42	0.32
HCMV reads (no.)	365,711	261,202	1,141,561	460,559	287,305
HCMV reads (%)	79.12	3.69	94.56	85.21	74.41
Mean read length	284	196	289	288	283
Average coverage depth + std	453±128	224±73	1355±825	577±176	355±124
